# Topology and parameter data of thirteen non-natural amino acids for molecular simulations with CHARMM22

**DOI:** 10.1016/j.dib.2016.09.051

**Published:** 2016-10-06

**Authors:** Olujide O. Olubiyi, Birgit Strodel

**Affiliations:** a Institute of Complex Systems: Structural Biochemistry (ICS-6), Forschungszentrum Jülich GmbH, 52425 Jülich, Germany; b Institute of Theoretical and Computational Chemistry, Heinrich Heine University Düsseldorf, 40225 Düsseldorf, Germany

**Keywords:** CHARMM, Force field parameterization, Quantum mechanics, Molecular dynamics, Potential energy surface, Geometry optimization

## Abstract

In this article we provide a data package containing the topology files and parameters compatible with the CHARMM22 force field for thirteen non-natural amino acids. The force field parameters were derived based on quantum mechanical (QM) calculations involving geometry optimization and potential energy surface scanning at the HF 6-31G(*d*) and HF 6-311G(*d,p*) levels of theory. The resulting energy data points were fitted to mathematical functions representing each component of the CHARMM22 force field. Further fine-tuning of the parameters utilized molecular mechanics energies, which were iteratively calculated and compared to the corresponding QM values until the latter were satisfactorily reproduced. The final force field data were validated with molecular dynamics simulations in explicit solvent conditions.

**Specifications Table**

**Table:** 

Subject area	*Chemistry, Biophysics*
More specific subject area	*Computational Biochemistry, Computational Biophysics*
Type of data	*Figures, tables, text*
How data was acquired	*Quantum mechanics (QM) and all-atom molecular dynamics (MD) calculations*
Data format	*Raw, analyzed*
Experimental factors	*Software used: Spartan 10 for QM, NAMD for MD*
Experimental features	*CHARMM22 force field*
Data source location	*Institute of Complex Systems: Structural Biochemistry (ICS-6), Forschungszentrum Jülich GmbH, 52425 Jülich, Germany*
Data accessibility	*Data are supplied with this article*

**Value of the data**

• New parameters for MD simulations of thirteen non-natural amino acids are provided.
• The parameters given here are compatible with the CHARMM22 force field, allowing to study the biophysical properties of these non-natural amino acids alone or as part of proteins and their interactions with other biomolecules and drugs. No further laborious parameterization is required.
• The employed parameterization approach provides a template for future design of hybrid amino acids, especially where it is desirable to combine small, drug-like organic molecular fragments with amino acid backbones either in the L or D configuration.

## 1 Data

**Fig. 1:**
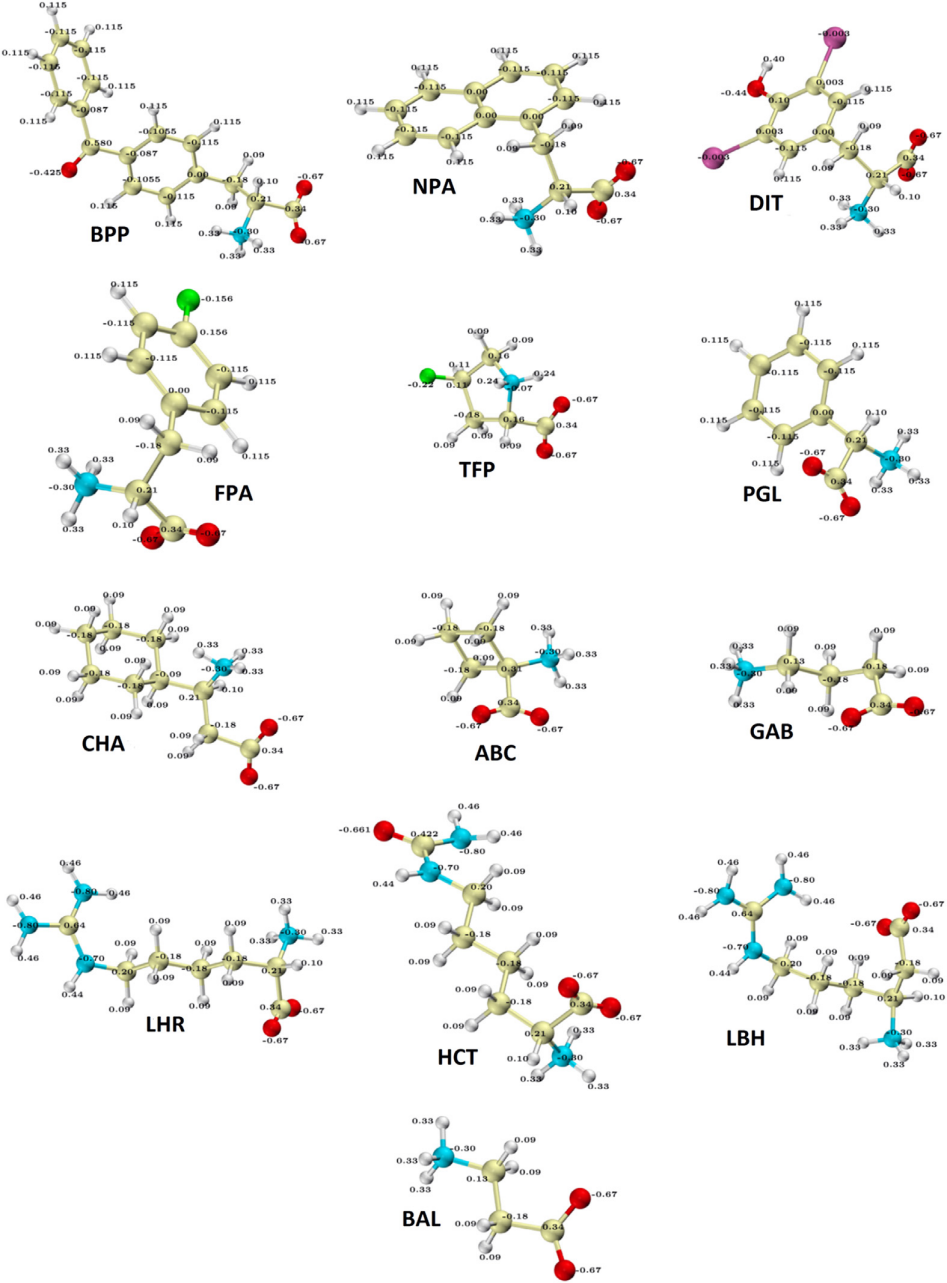
The chemical structures and atomic charges for the thirteen non-natural amino acids. Carbon atoms are shown in tan, nitrogen in blue, oxygen in red, hydrogen in white, fluorine in green and iodine in violet. See Fig. S1 in Supplementary Data for the atom labels.

In Supplementary material we provide the CHARMM22 topology and parameter files for following thirteen non-natural amino acids: D-4-fluo-rophenylalanine (FPA), D-4-benzoylphenylalanine (BPP), D-3,5-diiodotyrosine (DIT), γ-aminobutyric acid (GAB), D-cyclohexyl-β-alanine (CHA), D-phenylglycine (PGL), L-β-homoarginine (LBH), L-homoarginine (LHR), L-homocitrulline (HCT),D-4-transfluoroproline (TFP), D-aminocyclobutyl-carboxylic acid (ABC), β-alanine (BAL) and D-1-naphthylalanine (NPA). The chemical structures of these amino acids are shown in Fig. 1.

## 2 Experimental design, materials and methods

### 2.1 Parameterization

In the present work, we provide the topologies and CHARMM22 force field [1] parameters for thirteen non-natural amino acids to be used for molecular dynamics simulations. The parameterization process involved determining the equilibrium values for bond lengths, bond angles and dihedral angles and the force constants or energy barriers for the respective motion in case that these values were not already available in the CHARMM22 parameter set. To this end, QM calculations were performed for the thirteen amino acids using the program Spartan 10 [2]. The QM calculations were applied to the whole target molecules rather than to smaller representative submolecular systems as this eliminates the need for an extrapolation of the physicochemical properties of the amino acids from smaller model compounds. After generating structural models with the appropriate chirality, geometry optimization was performed at the Hartree–Fock (HF) 6-31G(*d*) level of theory using an optimization scheme combining the Geometric Direct Minimization method [3] and the Pulay Direct Inversion in the Iterative Subspace algorithm [4], [5]. A 3.0×10^−4^ hartrees/Bohr force tolerance was used. In the case of DIT, the HF 6-311G(*d,p*) basis set was employed as it allows higher flexibility for treating period V elements like iodine. The decision to perform the QM calculations at the HF 6-31G(*d*) level was based on the desire to stay close to the level of theory employed in parameterizing the CHARMM22 force field for the standard amino acids and also nucleotides.

After geometry optimization, QM potential energy scans were performed along the various bond stretching, angle bending and bond rotation coordinates. In the case of bond stretching, the potential energy was computed for twenty configurations uniformly spread between *b*_0_±0.25 Å with *b*_0_ as the equilibrium bond length. Similarly, energy profiles for the valence angles within the range *θ*_0_±5 were generated with *θ*_0_ as equilibrium bond angle. In the case of torsion, the full rotation −180°≤*δ*<180° was considered and potential energies calculated every 18°. The force constants were then calculated for bond stretching and angle bending by fitting the obtained potential energy data to harmonic functions using bond length and valence angle with the lowest energy along the potential energy curve as the equilibrium values, *b*_0_ and *θ*_0_, respectively. The energy barriers for bond torsions were determined by fitting the corresponding potential energy curve to a cosine function using the multiplicity as obtained from the energy scan.

**Fig. 2:**
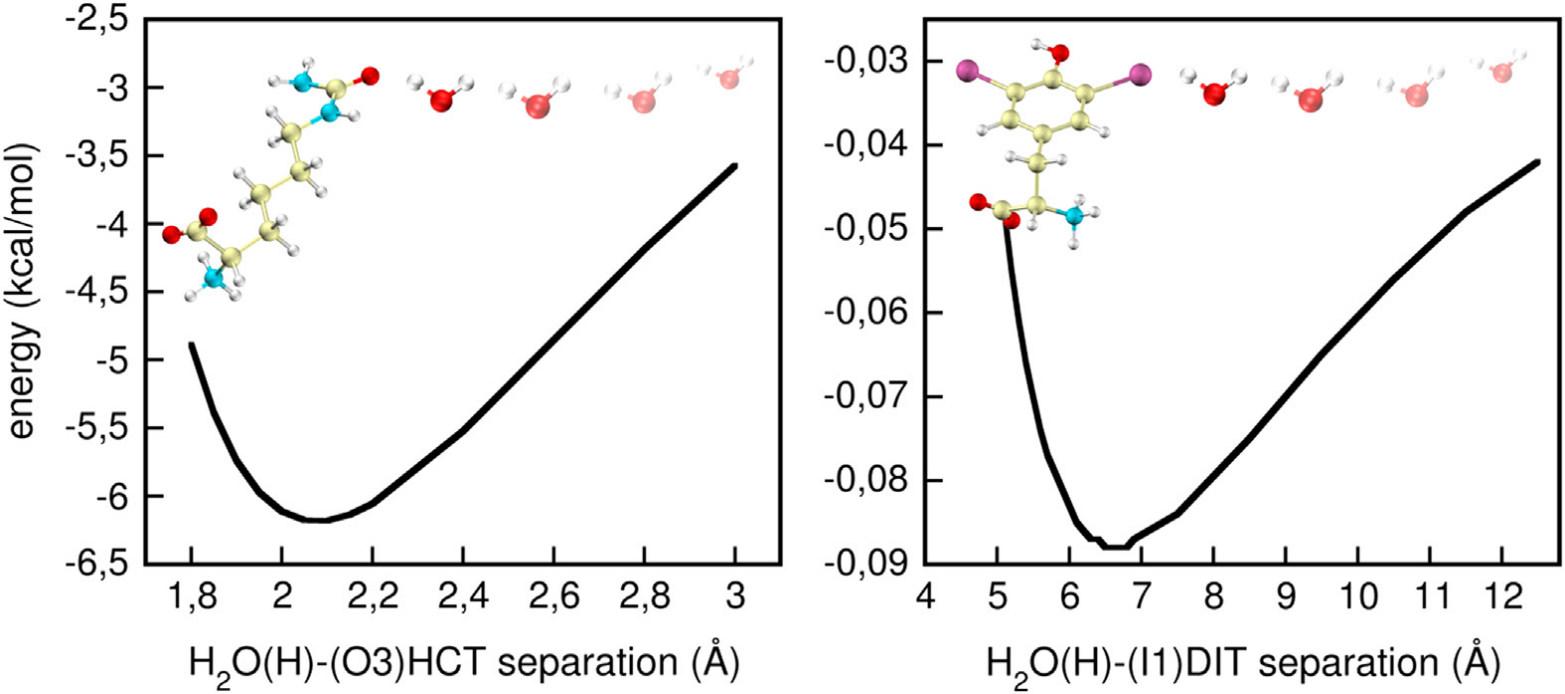
Potential energy for supramolecular HCT-water and DIT-water complexes. The change of interaction energy for different separations between the closest H atom of water and the atom of interest, i.e., atom O3 in HCT and atom I1 in DIT is shown. The structures at the energy minima were used for the Mulliken population analysis for deriving atomic charges in HCT and DIT.

For determining partial charges, original CHARMM22 values were taken, whenever possible, from similar atoms in a comparable local chemical environment. For instance, partial charges for the aromatic side chain of phenylalanine were taken for the aromatic atoms of PGL. Also, no new van der Waals (vdW) parameters had to be derived as for all atoms those already existing in the force field were used as no new atom types had to be defined. This approach, while avoiding the duplication of efforts ensures the new parameters to be close to—and thus compatible with— the existing CHARMM22 force field parameters. Only for few atoms, such as for atoms C7 and O3 of HCT and I1 of DIT (see Fig. S1 in Supplementary Data for the assignment of atom labels), charges had to be derived for which a method similar to that reported in Ref. [1] was employed. With this approach charges were taken from the Mulliken populations at the minimum of an interaction energy curve of a single water molecule [HF 6-31G(*d*)-optimized] forming a supramolecular complex with HCT [HF 6-31G(d)-optimized] via atom O3, and with DIT [HF 6-311G(*d,p*)-optimized] via atom I1. Starting with HCT-water and DIT-water supramolecular complexes, where water was separated by ~1.8 Å from the atom of interest, the water molecule in each case was systematically pulled away and the interaction energy calculated at selected separation distances. The resulting potential energy scan yielded the optimum molecule-water separations (2.15 Å and 6.6 Å in HCT and DIT, respectively) used for calculating the atomic charges by a Mulliken population analysis (Fig. 2).

The bonded parameters and atomic charges derived for all thirteen non-natural amino acids can be found in Tables S1–S4 in Supplementary data.

### 2.2 Validation

**Fig. 3:**
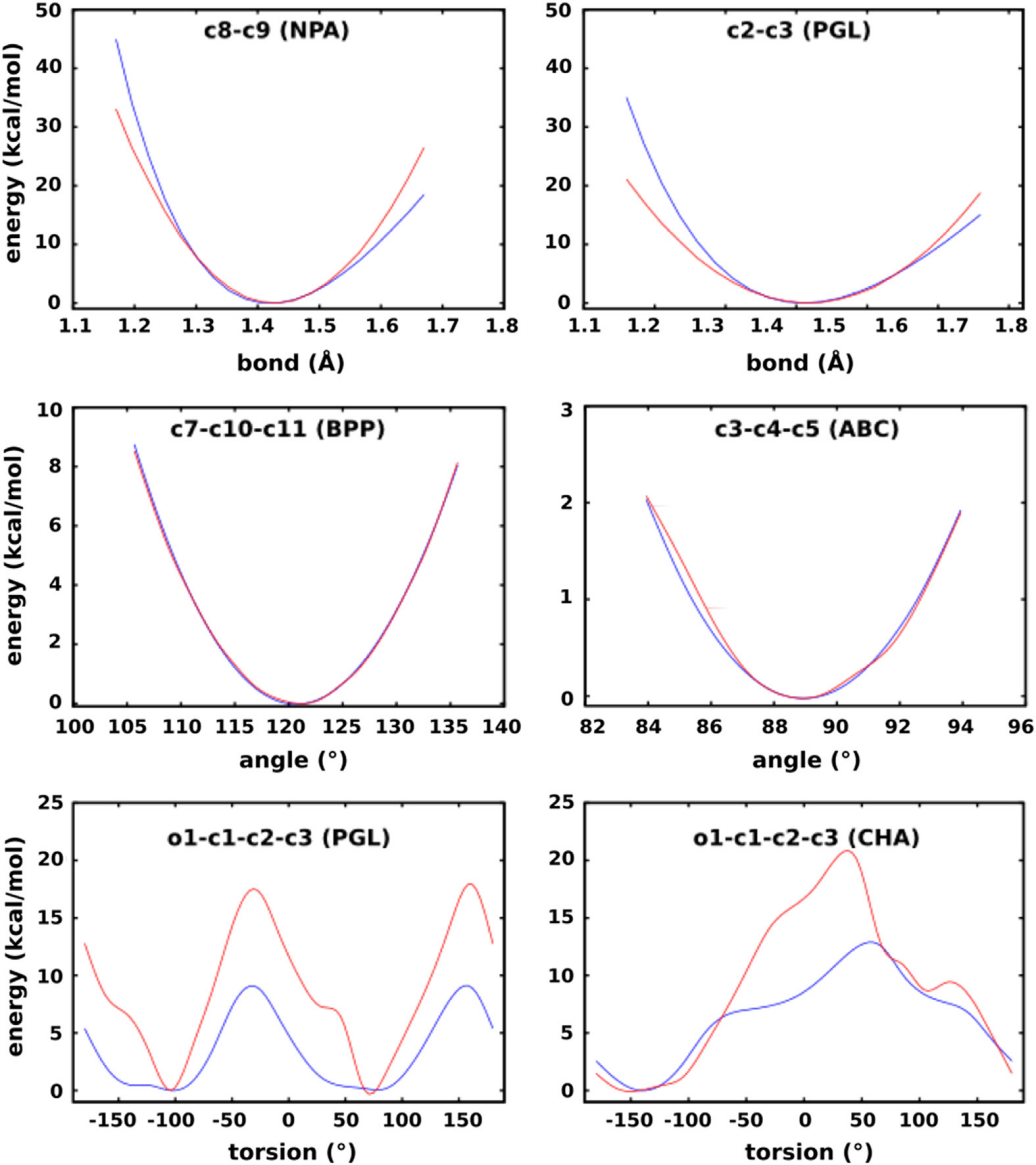
Representative potential energy curves for bond vibrations, angle bending and bond torsion (blue: QM; red: MM). See Figs. S1 and S2 in Supplementary Data for the assignment of atom labels.

To assess how well the newly derived force field parameters reproduce the QM energies, molecular mechanics (MM) energy scans were performed. Some of the resulting MM potential energy curves, after fine-tuning of parameters wherever necessary, superposed on the QM surfaces are presented in Fig. 3. The other 20 curves for the bonds and 44 curves for the angles are of similar quality (and are available upon request from the authors). The reproduction of the energy profile for the torsional motion is not as straightforward as it is for bonds and angles: To check how the energy overestimation compared with original force field parameters, equivalent torsional energy scans using existing CHARMM22 parameters were performed. The energy minima for the O1–C2–C3–C4 torsion in CHA correctly reproducing the QM minima but overestimating the barrier height by about 7 kcal/mol, are presented in Fig. 3.

For further validation of the applicability of the newly derived MM parameters, they were subsequently employed in 1 ns MD simulations of each of the thirteen non-natural amino acids treated as a zwitterion and immersed in a cube of TIP3P water at 300 K and 1 bar. A 12 Å cut-off was used for the calculation of short-range non-bonded interactions and periodic boundary conditions employed for boundary treatment in connection with the particle mesh Ewald method for calculating Coulomb interactions. A Langevin thermostat was used for temperature control and a Nosé-Hoover piston barostat for pressure control. The solvated systems were subjected to 1000 steps of energy minimization after which the MD runs were performed using a time step of 1 fs. System preparation was done in VMD [7] while both the energy minimization and MD steps were carried out using the NAMD [8] simulation program. The MD simulations were employed to obtain insight into the degree of structural stability to expect in typical MD simulations employing the parameters. To this end, root mean square deviation (RMSD) calculations were performed after least square fitting to the heavy atoms.

## Footnotes

Transparency document Transparency data associated with this article can be found in the online version at doi:10.1016/j.dib.2016.09.051.
