# Supplementary material for: Topology and parameter data of thirteen non-natural amino acids for molecular simulations with CHARMM22
Source: Data Brief. 2016 Oct 6;9:642–7. doi: 10.1016/j.dib.2016.09.051 (PMC5067094; doi:10.1016/j.dib.2016.09.051)
Supplement: Supplementary file 1 — Supplementary material [file mmc1.docx]

Conflicts of interest: None

The authors have no conflicts of interest to declare.
