# Supplementary material for: Topology and parameter data of thirteen non-natural amino acids for molecular simulations with CHARMM22
Source: Data Brief. 2016 Oct 6;9:642–7. doi: 10.1016/j.dib.2016.09.051 (PMC5067094; doi:10.1016/j.dib.2016.09.051)
Supplement: Supplementary file 2 — Supplementary material [file mmc2.docx]

**Supplementary Data**

**Topology and parameter data of thirteen non-natural amino acids for molecular simulations with CHARMM22**

***Olujide O Olubiyi^1,2^, and Birgit Strodel^1,3^**

Correspondence to: Olujide O. Olubiyi (E-mail: [*olubiyioo@abuad.edu.ng*](mailto:olubiyioo@abuad.edu.ng))

1*Institute of Complex Systems: Structural Biochemistry (ICS-6), Forschungszentrum Jülich GmbH, 52425 Jülich, Germany*

2 *Author now works at Department of Pharmacology & Therapeutics, College of Medicine & Health Sciences, Afe Babalola University Nigeria*

*^3^Institut of Theoretical and Computational Chemistry, Heinrich Heine University Düsseldorf, 40225 Düsseldorf, Germany*

**1) Supplementary Figures**


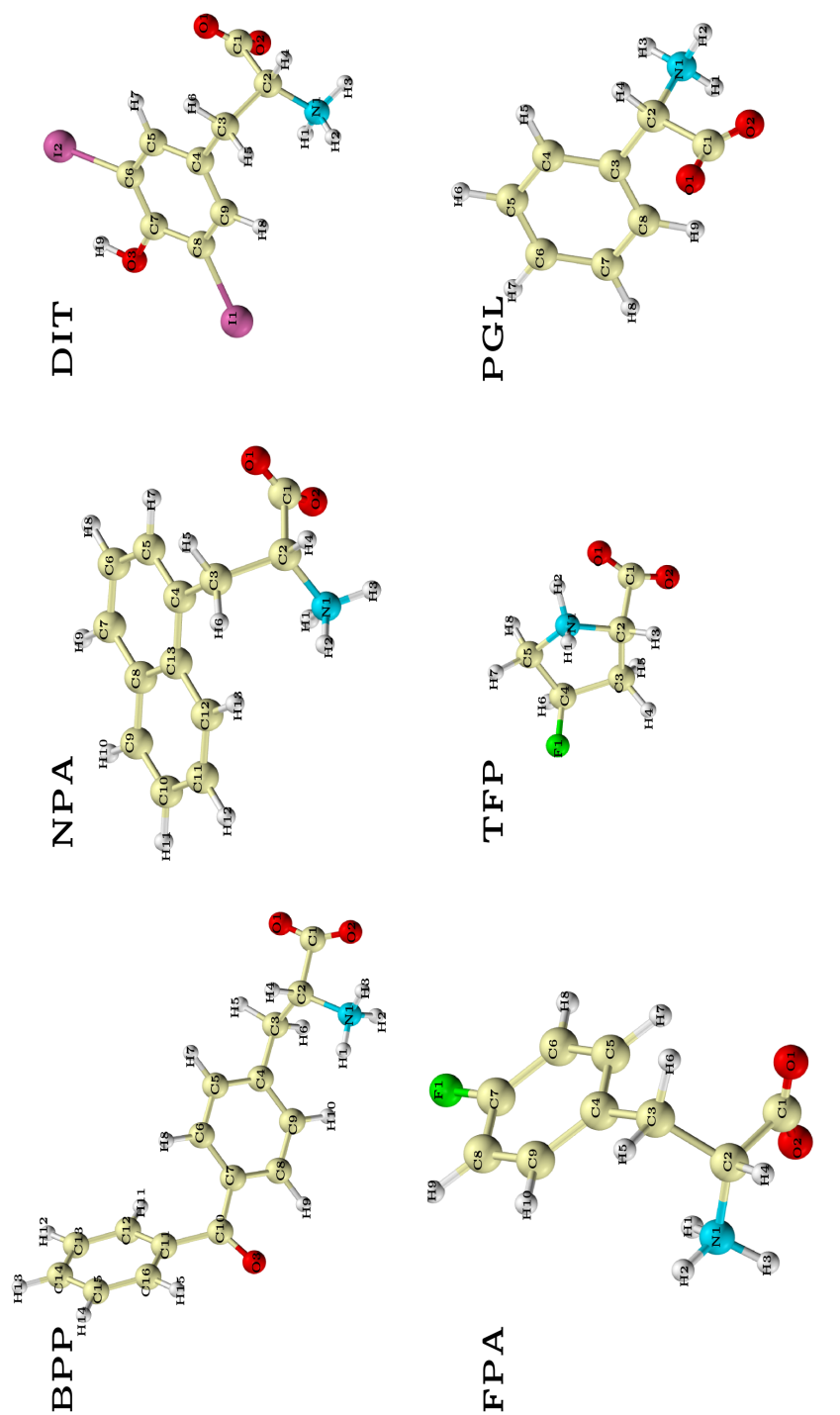


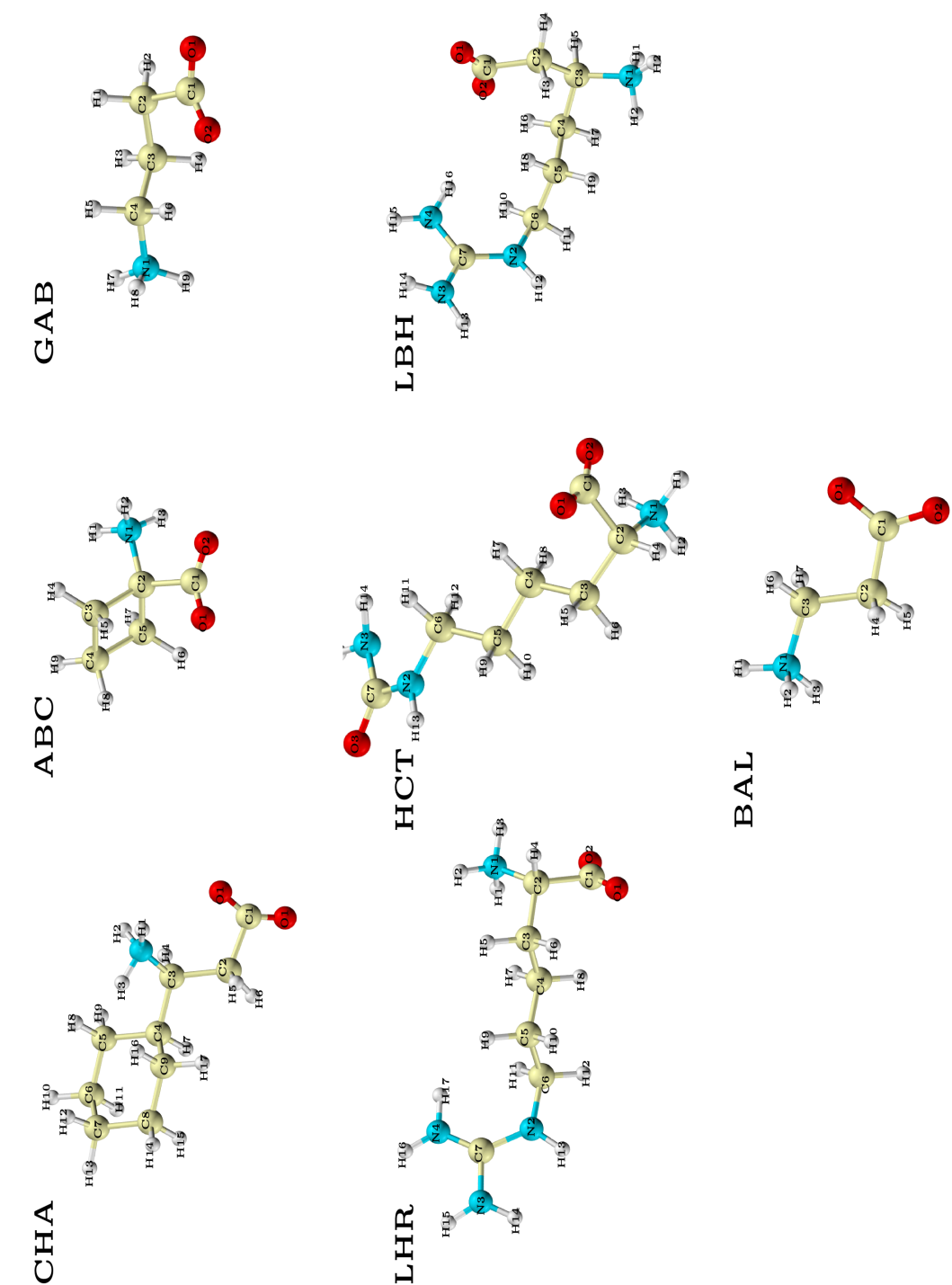


**Fig. S1.** Atomic labels for the non-natural amino acids parameterized in this work.

**2) Supplementary Tables**

| TABLE S1. Calculated bond parameters. | | | | | | | |
| --- | --- | --- | --- | --- | --- | --- | --- |
| **AA^[a]^** | **Bond^[b]^** | ***b*_0_^[c]^** | ***k_b_*^[d]^** | **AA^[a]^** | **Bond^[b]^** | ***b*_0_^[c]^** | ***k_b_*^[d]^** |
| ABC | C2-C3 | 1.553 | 304.4 | CHA | C4-C5 | 1.537 | 273.4 |
| ABC | C3-C4 | 1.552 | 338.0 | CHA | C4-H7 | 1.103 | 317.8 |
| ABC | H4-C3 | 1.094 | 402.3 | DIT | C8-I1 | 2.116 | 156.1 |
| BAL | C2-C3 | 1.510 | 255.0 | FPA | F1-C7 | 1.308 | 427.2 |
| BAL | C2-H4 | 1.087 | 288.4 | HCT | N2-C7 | 1.372 | 395.8 |
| BAL | C3-H6 | 1.076 | 372.6 | HCT | O3-C7 | 1.201 | 739.5 |
| BPP | C10-C11 | 1.505 | 395.4 | NPA | C13-C8 | 1.425 | 419.3 |
| BPP | O3-C10 | 1.211 | 880.5 | NPA | C8-C9 | 1.433 | 432.5 |
| CHA | C3-C4 | 1.544 | 263.5 | NPA | C11-H12 | 1.075 | 383.3 |
| CHA | C5-C6 | 1.532 | 280.4 | PGL | C3-C2 | 1.510 | 310.1 |
| CHA | C5-H8 | 1.101 | 326.0 | TFP | F1-C4 | 1.381 | 312.5 |
| ^[a]^Amino acid. ^[b]^Atom labels employed are in accordance with figure S1 and the topologies presented below. ^[c]^Equilibrium bond length in Å. ^[d]^Force constant in kcal/mol/Å^2^ | | | | | | | |

| TABLE S2. Computed valence angle parameters. | | | | | | | | | | | | | | | | |
| --- | --- | --- | --- | --- | --- | --- | --- | --- | --- | --- | --- | --- | --- | --- | --- | --- |
| **AA^[a]^** | | **Angle^[b]^** | | ***θ*_0_^[c]^** | | ***k_θ_*^[d]^** | | | **AA^[a]^** | | **Angle^[b]^** | ***θ*_0_^[c]^** | | | ***k_θ_*^[d]^** | |
| ABC | | C2-C3-C4 | | 89.120 | | 90.8 | | | CHA | | H8-C5-H9 | 106.740 | | | 59.4 | |
| ABC | | C2-C3-H4 | | 116.080 | | 60.5 | | | CHA | | H9-C5-C4 | 109.780 | | | 68.3 | |
| ABC | | C3-C4-C5 | | 88.940 | | 130.1 | | | CHA | | H7-C4-C5 | 107.030 | | | 72.7 | |
| ABC | | C5-C2-C1 | | 110.350 | | 104.2 | | | CHA | | N1-C3-C4 | 115.850 | | | 119.0 | |
| ABC | | C5-C2-C3 | | 88.000 | | 382.2 | | | DIT | | C9-C8-I1 | 118.770 | | | 121.7 | |
| ABC | | H4-C3-C4 | | 117.110 | | 59.1 | | | FPA | | C8-C7-F1 | 119.860 | | | 126.1 | |
| ABC | | H4-C3-H5 | | 109.580 | | 57.8 | | | HCT | | C7-N3-H14 | 111.150 | | | 45.9 | |
| ABC | | H5-C3-C4 | | 111.760 | | 56.6 | | | HCT | | N2-C7-N3 | 115.740 | | | 138.0 | |
| ABC | | N1-C2-C1 | | 111.430 | | 350.0 | | | HCT | | O3-C7-N3 | 122.040 | | | 140.8 | |
| ABC | | N1-C2-C3 | | 118.000 | | 200.0 | | | NPA | | C3-C4-C13 | 122.380 | | | 134.3 | |
| BAL | | C3-C2-C1 | | 108.630 | | 98.3 | | | NPA | | C8-C13-C12 | 118.030 | | | 220.9 | |
| BAL | | H4-C2-H5 | | 107.720 | | 57.5 | | | NPA | | C8-C7-C6 | 120.610 | | | 202.6 | |
| BAL | | H6-C3-H7 | | 109.210 | | 57.4 | | | NPA | | C8-C7-H9 | 118.830 | | | 79.8 | |
| BAL | | N1-C3-C2 | | 109.720 | | 88.6 | | | NPA | | C7-C8-C9 | 120.660 | | | 171.9 | |
| BPP | | C7-C10-C11 | | 121.000 | | 55.0 | | | PGL | | C3-C2-C1 | 113.670 | | | 80.9 | |
| BPP | | O3-C10-C11 | | 120.500 | | 100.8 | | | PGL | | C3-C2-H4 | 111.100 | | | 72.6 | |
| CHA | | C2-C3-C4 | | 112.440 | | 113.0 | | | PGL | | C3-C2-N1 | 109.380 | | | 97.9 | |
| CHA | | C3-C4-C5 | | 117.700 | | 116.0 | | | PGL | | C4-C3-C2 | 120.610 | | | 119.5 | |
| CHA | | C3-C4-H7 | | 105.740 | | 72.6 | | | TFP | | F1-C4-C3 | 108.580 | | | 102.2 | |
| CHA | | C5-C6-C7 | | 111.350 | | 118.6 | | | TFP | | F1-C4-C5 | 108.120 | | | 99.9 | |
| CHA | | C4-C5-C6 | | 111.900 | | 117.3 | | | TFP | | F1-C4-H6 | 107.490 | | | 85.6 | |
| CHA | | H4-C3-H4 | | 106.570 | | 76.6 | | | TFP | | H6-C4-C3 | 114.310 | | | 67.6 | |
| CHA | | H8-C5-C4 | | 109.840 | | 67.4 | | | TFP | | H6-C4-F1 | 115.050 | | | 66.0 | |
| ^[a]^Amino acid. ^[b]^Atom labels employed are in accordance with figure S1 and the topologies presented below. ^[c]^Equilibrium angle in degree. ^[d]^Force constant in kcal/mol/rad^2^. | | | | | | | | | | | | | | | | |
| TABLE S3. Dihedral angle parameters. | | | | | | | | | | | | | | | | |
| **AA^[a^** | **Dihedral^[b]^** | | ***δ*_0_^[c]^** | | ***V_δ_*^[d]^** | | ***m*^[e]^** | **AA^[a]^** | | **Dihedral^[b]^** | | | ***δ*_0_^[c]^** | ***V_δ_*^[d]^** | | ***m*^[e]^** |
| ABC | C3-C2-C1-O1 | | -129.4 | | 3.844 | | 2 | CHA | | X-C4-C5-X | | | 0.0 | 0.157 | | 3 |
| ABC | X- C3-C4-X* | | 0.0 | | 0.160 | | 3 | CHA | | X-C5-C6-X | | | 0.0 | 0.157 | | 3 |
| ABC | H1-N1-C2-C1 | | -123.1 | | 0.637 | | 1 | DIT | | C4-C9-C8-I1 | | | 180.0 | 2.100 | | 2 |
| ABC | H1-N1-C2-C1 | | 0.0 | | 0.600 | | 1 | DIT | | I1-C8-C9-H8 | | | 180.0 | 3.000 | | 2 |
| ABC | H1-N1-C2-C1 | | 123.1 | | 0.637 | | 1 | DIT | | I1-C8-C7-O3 | | | 180.0 | 3.000 | | 2 |
| ABC | H1-N1-C2-C3 | | 0.0 | | 0.198 | | 3 | FPA | | F1-C7-C6-C5 | | | 180.0 | 55.113 | | 2 |
| ABC | N1-C2-C1-O1 | | 177.6 | | 4.618 | | 2 | FPA | | F1-C7-C6-H8 | | | 180.0 | 6.087 | | 2 |
| ABC | C'-C1'-N1-C2 | | 180.0 | | 2.750 | | 2 | GAB | | H1-N1-C4-C3 | | | 0.0 | 1.321 | | 3 |
| ABC | X-C2-C3-X | | 0.0 | | 0.140 | | 3 | GAB | | C3-C2-C1-O1 | | | 0.0 | 0.335 | | 2 |
| BAL | N1-C3-C2-C1 | | 0.0 | | 12.177 | | 2 | GAB | | C3-C2-C1-O1 | | | 0.0 | 0.938 | | 1 |
| BAL | C3-C2-C1-O1 | | 0.0 | | 0.335 | | 2 | GAB | | X-C3-C2-X | | | 0.0 | 0.195 | | 3 |
| BAL | H4-C2-C3-H6 | | -47.4 | | 4.054 | | 3 | GAB | | X-C3-C4-X | | | 0.0 | 0.195 | | 3 |
| BAL | H6-C3-C2-C1 | | -161.1 | | 3.829 | | 4 | HCT | | C6-N2-C7-O3 | | | 9.5 | 2.211 | | 4 |
| BAL | N1-C3-C2-H4 | | 0.0 | | 3.521 | | 4 | HCT | | N2-C7-N3-H14 | | | 28.7 | 2.465 | | 1 |
| BAL | H1-N1-C3-C2 | | 0.0 | | 1.321 | | 3 | HCT | | N2-C7-N3-H14 | | | -28.4 | 2.982 | | 1 |
| BAL | H1-N1-C3-H6 | | 0.0 | | 1.312 | | 3 | HCT | | N2-C7-N3-H14 | | | -180.0 | 2.926 | | 1 |
| BAL | H4-C2-C1-O1 | | -123.1 | | 1.131 | | 2 | HCT | | N2-C7-N3-H14 | | | 180.0 | 2.926 | | 1 |
| BPP | C8-C7-C10-C11 | | -28.4 | | 1.538 | | 5 | HCT | | H14-N3-C7-O3 | | | -143.2 | 10.829 | | 1 |
| BPP | C8-C7-C10-O3 | | 28.4 | | 0.800 | | 4 | HCT | | H14-N3-C7-O3 | | | 9.5 | 10.678 | | 1 |
| BPP | H9-C8-C7-C10 | | 180.0 | | 0.000 | | 2 | HCT | | H14-N3-C7-O3 | | | 142.1 | 3.348 | | 1 |
| BPP | C9-C8-C7-C10 | | -180.0 | | 0.000 | | 1 | HCT | | C6-N2-C7-N3 | | | 180.0 | 2.250 | | 2 |
| CHA | N1-C3-C4-H7 | | 0.0 | | 2.934 | | 3 | PGL | | C1-C2-C3-C4 | | | -143.2 | 0.723 | | 3 |
| CHA | N1-C3-C4-C5 | | 66.3 | | 2.193 | | 3 | PGL | | H4-C2-C3-C4 | | | -9.5 | 0.784 | | 3 |
| CHA | HA-C3-C4-H7 | | 66.3 | | 2.089 | | 3 | PGL | | H1-N1-C2-C3 | | | 104.2 | 0.575 | | 6 |
| CHA | HA-C3-C4-C5 | | -47.4 | | 2.391 | | 3 | PGL | | N1-C2-C3-C4 | | | 104.2 | 0.717 | | 2 |
| CHA | C2-C3-C4-H7 | | -47.4 | | 2.383 | | 3 | PGL | | C2-C3-C4-C5 | | | 180.0 | 3.100 | | 2 |
| CHA | C2-C3-C4-C5 | | 0.0 | | 2.689 | | 3 | PGL | | X-C2-C1-X | | | 180.0 | 0.000 | | 6 |
| CHA | N1-C1-C2-C3 | | 0.0 | | 0.000 | | 1 | PGL | | X-C2-N1-X | | | 0.0 | 0.100 | | 3 |
| CHA | O1-C1-C2-C3 | | 0.0 | | 1.400 | | 1 | PGL | | H1-N1-C2-C3 | | | 104.2 | 0.575 | | 3 |
| CHA | O1-C1-C2-C3 | | -142.1 | | 2.507 | | 1 | PGL | | H5-C3-C4-C2 | | | -104.2 | 0.000 | | 3 |
| ^[a]^Amino acid. ^[b]^ Atom labels employed are in accordance with figure S1 and the topologies presented below. ^[c]^Torsion angle in degree. ^[d]^ Torsional potential in kcal/mol. ^[e]^Multiplicity. ^[^*^]^X stands for any of the commonly encountered atoms. | | | | | | | | | | | | | | | | |

| TABLE S4. Atomic charges | | | |
| --- | --- | --- | --- |
| **Element** | **Charge** | **Description** | **Amino acid** |
| C | +0.340 | C-terminal carboxylate carbon | All |
| C | +0.580 | Carboxylate carbon of diphenyl ketone | BPP |
| C | +0.422 | Carboxylate carbon with diamino groups | HCT |
| C | +0.310 | Tertiary sp3 carbon bonded to nitrogen | ABC |
| C | -0.180 | Secondary sp3 carbon | All^[a]^ |
| C | -0.115 | Protonated aromatic carbon | BPP, NPA, DIT, FPA, PGL |
| C | -0.000 | Non-protonated aromatic carbon | BPP, NPA, DIT, FPA, PGL |
| C | -0.087 | Non-protonated aromatic carboxylate bonded carbon. | BPP |
| C | +0.100 | Hydroxylated aromatic carbon | DIT |
| C | +0.003 | Iodinated aromatic carbon | DIT |
| C | +0.003 | Iodinated aromatic carbon | DIT |
| C | +0.160 | N-bonded secondary sp3 carbon of fluorinated proline | TFP |
| C | +0.110 | Fluorinated sp3 carbon | TFP |
| N | -0.300 | N-terminal ammonium nitrogen | All |
| N | -0.070 | Proline nitrogen | TFP |
| N | -0.700 | Guanidinium singly protonated nitrogen | HCT, LBH, LHR |
| N | -0.800 | Guanidinium doubly protonated nitrogen | HCT, LBH, LHR |
| O | -0.670 | C-terminal carboxylate oxygen | All |
| O | -0425 | Carboxylate oxygen in diphenyl ketone | BPP |
| O | -0.661 | Carboxylate oxygen of diamino ketone | HCT |
| O | -0.440 | Phenolic oxygen | DIT |
| H | +0.330 | N-terminal ammonium hydrogen | All |
| H | +0.240 | Terminal hydrogen on proline nitrogen | TFP |
| H | +0.440 | Hydrogen singly protonating nitrogen in guanidinium system | HCT, LBH, LHR |
| H | +0.460 | Hydrogen doubly protonating nitrogen in guanidinium system | HCT, LBH, LHR |
| H | +0.110 | Hydrogen on fluorinated sp3 carbon | TFP |
| H | +0.090 | Polar hydrogen atoms | All |
| F | -0.156 | Fluorine of fluorinated aromatic ring | FPA |
| F | -0.220 | Fluorine of fluorinated aliphatic ring | TFP |
| I | -0.003 | Iodine of iodinated aromatic ring | DIT |
| ^[a]^All amino acids with the exception of PGL. | | | |

**3) Topologies in CHARMM22 format**

**RESI LHR 1.00 ! L-Homoarginine**

GROUP

ATOM N NH1 -0.47 ! | HH11

ATOM HN H 0.31 ! HN-N |

ATOM CA CT1 0.07 ! | HB1 HG1 HD1 HE1 HZ NH1-HH12

ATOM HA HB 0.09 ! | | | | | | //(+)

GROUP ! HA-CA--CB--CG--CD--CE--NZ--CI

ATOM CB CT2 -0.18 ! | | | | | \

ATOM HB1 HA 0.09 ! | HB2 HG2 HD2 HE2 NH2-HH22

ATOM HB2 HA 0.09 ! O=C |

GROUP ! | HH21

ATOM CG CT2 -0.18

ATOM HG1 HA 0.09

ATOM HG2 HA 0.09

GROUP

ATOM CD CT2 -0.18

ATOM HD1 HA 0.09

ATOM HD2 HA 0.09

GROUP

ATOM CE CT2 0.20

ATOM HE1 HA 0.09

ATOM HE2 HA 0.09

ATOM NZ NC2 -0.70

ATOM HZ HC 0.44

ATOM CI C 0.64

ATOM NH1 NC2 -0.80

ATOM HH11 HC 0.46

ATOM HH12 HC 0.46

ATOM NH2 NC2 -0.80

ATOM HH21 HC 0.46

ATOM HH22 HC 0.46

GROUP

ATOM C C 0.51

ATOM O O -0.51

BOND CB CA CG CB CD CG CE CD NZ CE CI NZ

BOND NH2 CI N HN N CA

BOND C CA C +N CA HA CB HB1

BOND CB HB2 CG HG1 CG HG2 CD HD1 CD HD2 CE HE1 CE HE2

BOND NZ HZ NH1 HH11 NH1 HH12 NH2 HH21 NH2 HH22

DOUBLE O C CI NH1

IMPR N -C CA HN C CA +N O

CMAP -C N CA C N CA C +N

IMPR CI NH1 NH2 NZ

DONOR HN N

DONOR HZ NZ

DONOR HH11 NH1

DONOR HH12 NH1

DONOR HH21 NH2

DONOR HH22 NH2

ACCEPTOR O C

IC -C CA *N HN 1.3496 122.4500 180.0000 116.6700 0.9973

IC -C N CA C 1.3496 122.4500 180.0000 109.8600 1.5227

IC N CA C +N 1.4544 109.8600 180.0000 117.1200 1.3511

IC +N CA *C O 1.3511 117.1200 180.0000 121.4000 1.2271

IC CA C +N +CA 1.5227 117.1200 180.0000 124.6700 1.4565

IC N C *CA CB 1.4544 109.8600 123.6400 112.2600 1.5552

IC N C *CA HA 1.4544 109.8600 117.9300 106.6100 1.0836

IC N CA CB CG 1.4544 110.7000 180.0000 115.9500 1.5475

IC CG CA *CB HB1 1.5475 115.9500 120.0500 106.4000 1.1163

IC CG CA *CB HB2 1.5475 115.9500 -125.8100 109.5500 1.1124

IC CA CB CG CD 1.5552 115.9500 180.0000 114.0100 1.5384

IC CD CB *CG HG1 1.5384 114.0100 125.2000 108.5500 1.1121

IC CD CB *CG HG2 1.5384 114.0100 -120.3000 108.9600 1.1143

IC CB CG CD CE

IC CE CG *CD HD1

IC CE CG *CD HD2

IC CG CD CE NZ 1.5475 114.0100 180.0000 107.0900 1.5034

IC NZ CD *CE HE1 1.5034 107.0900 120.6900 109.4100 1.1143

IC NZ CD *CE HE2 1.5034 107.0900 -119.0400 111.5200 1.1150

IC CD CE NZ CI 1.5384 107.0900 180.0000 123.0500 1.3401

IC CI CE *NZ HZ 1.3401 123.0500 180.0000 113.1400 1.0065

IC CE NZ CI NH1 1.5034 123.0500 180.0000 118.0600 1.3311

IC NZ CI NH1 HH11 1.3401 118.0600 -178.2800 120.6100 0.9903

IC HH11 CI *NH1 HH12 0.9903 120.6100 171.1900 116.2900 1.0023

IC NH1 NZ *CI NH2 1.3311 118.0600 178.6400 122.1400 1.3292

IC NZ CI NH2 HH21 1.3401 122.1400 -174.1400 119.9100 0.9899

IC HH21 CI *NH2 HH22 0.9899 119.9100 166.1600 116.8800 0.9914

**RESI CHA +0.00 ! D-Betacyclohexylalanine**

GROUP ! HD12 HE12

ATOM N NH1 -0.47 ! | \HD11 HE11/

ATOM HN H 0.31 ! O=C \ / \ /

ATOM CB CT1 0.07 ! | HB CD1--CE1 HZ1

ATOM HB HA 0.09 ! | | / \ /

GROUP ! HA1--CA--CB----CG--HG CZ

ATOM CA CT2 -0.18 ! | | \ / \

ATOM HA1 HB 0.09 ! HA2 | CD2--CE2 HZ2

ATOM HA2 HB 0.09 ! | / \ / \

GROUP ! | /HD21 HE21\

ATOM CG CHX1 -0.09 ! HN--N HD22 HE22

ATOM HG HHX1 0.09 ! |

GROUP ! |

ATOM CD1 CHX2 -0.18 !

ATOM HD11 HHX2 0.09 !

ATOM HD12 HHX2 0.09 !

GROUP !

ATOM CD2 CHX2 -0.18 !

ATOM HD21 HHX2 0.09 !

ATOM HD22 HHX2 0.09 !

GROUP !

ATOM CE1 CHX2 -0.18 !

ATOM HE11 HHX2 0.09 !

ATOM HE12 HHX2 0.09 !

GROUP !

ATOM CE2 CHX2 -0.18 !

ATOM HE21 HHX2 0.09 !

ATOM HE22 HHX2 0.09 !

GROUP !

ATOM CZ CHX2 -0.18 !

ATOM HZ1 HHX2 +0.09 !

ATOM HZ2 HHX2 +0.09 !

GROUP !

ATOM C C +0.51 !

ATOM O O -0.51 !

BOND CB N HN N HB CB CA CB

BOND CG CB HA1 CA HA2 CA C CA

BOND O C CD1 CG CD2 CG HG CG

BOND CZ CE2 CD2 CE2 HE21 CE2 HE22 CE2

BOND CZ CE1 CD1 CE1 HE11 CE1 HE12 CE1

BOND HZ1 CZ HZ2 CZ HD11 CD1 HD12 CD1

BOND HD21 CD2 HD22 CD2 C +N

!CMAP -C N CB CA !CMAP CB CA C +N!CMAP N CB CA C

DONOR HN N

ACCEPTOR O C

IMPR N -C CB HN

IMPR C CA +N O

IMPR CB CG N CA

IC -C CB *N HN 1.348 123.81 180.00 114.54 0.999

IC +N CA *C O 1.348 117.33 180.00 120.67 1.229

IC CA C +N +CA 1.523 117.33 180.00 124.31 1.451

IC N CA *CB CG 1.450 106.52 -122.27 112.34 1.551

IC N CA *CB HB 1.450 106.52 118.89 109.12 1.126

IC CB C *CA HA1 1.540 113.64 -122.27 112.34 1.110

IC CB C *CA HA2 1.540 113.64 118.89 112.34 1.110

IC HA2 CA CB CG 1.112 110.07 41.50 112.44 1.551

IC HA1 CA CB CG 1.110 111.12 -74.32 112.44 1.551

IC HA2 CA CB HB 1.112 110.07 -76.57 108.21 1.126

IC HA1 CA CB HB 1.110 111.12 167.61 108.21 1.126

IC HA2 CA CB N 1.112 110.07 168.11 108.06 1.496

IC HA1 CA CB N 1.110 111.12 52.29 108.06 1.496

IC HA2 CA CB N 1.112 110.07 168.11 108.06 1.496

IC HA1 CA CB N 1.110 111.12 52.29 108.06 1.496

IC CG CA *CB HB 1.511 112.94 118.89 109.12 1.112

IC CG CA *CB N 1.511 112.94 -123.36 110.70 1.496

IC -C N CB CA 1.348 123.81 180.00 113.64 1.540

IC CB CA C +N 1.540 113.64 180.00 117.33 1.348

IC CG CB N HN 1.551 115.85 -67.54 110.74 1.017

IC HB CB N HN 1.126 106.82 51.85 110.74 1.017

IC CA CB N HN 1.540 108.06 168.08 110.74 1.017

IC C CA CB CG 1.514 113.64 162.99 112.44 1.551

IC C CA CB HB 1.514 113.64 44.92 108.21 1.126

IC C CA CB N 1.514 113.64 -70.40 108.06 1.496

IC CD1 CG CB HB 1.537 111.70 -47.38 107.79 1.126

IC CD2 CG CB HB 1.540 111.70 0.00 107.79 1.126

IC HG CG CB HB 1.103 105.74 66.29 107.79 1.126

IC CD1 CG CB N 1.537 110.55 66.29 115.85 1.496

IC CD2 CG CB N 1.540 113.02 -57.49 115.85 1.496

IC HG CG CB N 1.121 105.74 0.00 115.85 1.496

IC CD1 CG CB CA 1.537 111.70 0.00 112.44 1.540

IC CD2 CG CB CA 1.537 111.70 65.34 112.44 1.540

IC HG CG CB CA 1.103 105.74 -47.38 112.44 1.540

IC O C CA HA2 1.208 124.61 -60.57 108.17 1.112

IC O C CA HA1 1.208 124.61 60.57 108.48 1.110

IC O C CA CB 1.208 124.61 -134.30 113.64 1.540

IC HD11 CD1 CG CD2 1.101 109.84 0.00 110.25 1.537

IC HD12 CD1 CG CD2 1.101 109.84 64.11 110.25 1.537

IC CE1 CD1 CG CD2 1.532 111.90 -57.20 110.25 1.537

IC HD11 CD1 CG CB 1.101 109.84 55.55 110.55 1.551

IC HD12 CD1 CG CB 1.101 109.84 -61.59 110.55 1.551

IC CE1 CD1 CG CB 1.532 111.90 177.11 110.55 1.551

IC HD11 CD1 CG HG 1.101 109.84 -61.28 107.69 1.103

IC HD12 CD1 CG HG 1.101 109.84 0.00 107.69 1.103

IC CE1 CD1 CG HG 1.532 111.90 60.28 107.69 1.103

IC HD21 CD2 CG CD1 1.101 109.84 178.56 110.25 1.537

IC HD22 CD2 CG CD1 1.101 109.84 -64.25 110.25 1.537

IC CE2 CD2 CG CD1 1.532 111.90 57.09 110.25 1.537

IC HD21 CD2 CG CB 1.101 109.84 -57.15 113.02 1.551

IC HD22 CD2 CG CB 1.101 109.84 60.04 113.02 1.551

IC CE2 CD2 CG CB 1.532 111.90 0.00 113.02 1.551

IC HD21 CD2 CG HG 1.101 109.84 61.21 107.89 1.103

IC HD22 CD2 CG HG 1.101 109.84 178.39 107.89 1.103

IC CE2 CD2 CG HG 1.532 111.90 -60.27 107.89 1.103

IC HZ2 CZ CE2 HE22 1.101 109.93 54.96 109.81 1.101

IC HZ1 CZ CE2 HE22 1.101 109.83 171.21 109.81 1.101

IC CE1 CZ CE2 HE22 1.532 111.19 -67.02 109.81 1.101

IC HZ2 CZ CE2 CD2 1.101 109.93 176.88 111.35 1.532

IC HZ1 CZ CE2 CD2 1.101 109.83 -66.87 111.35 1.532

IC CE1 CZ CE2 CD2 1.532 111.19 54.91 111.35 1.532

IC HZ2 CZ CE2 HE21 1.101 109.93 -61.16 109.93 1.101

IC HZ1 CZ CE2 HE21 1.101 109.83 55.08 109.93 1.101

IC CE1 CZ CE2 HE21 1.532 111.19 176.86 109.93 1.101

IC CG CD2 CE2 CZ 1.537 111.90 -56.18 111.43 1.532

IC HD21 CD2 CE2 CZ 1.101 109.57 0.00 111.43 1.532

IC HD22 CD2 CE2 CZ 1.101 109.32 65.85 111.43 1.532

IC CG CD2 CE2 HE22 1.537 111.90 65.73 109.84 1.101

IC HD21 CD2 CE2 HE22 1.101 109.57 -56.15 109.84 1.101

IC HD22 CD2 CE2 HE22 1.101 109.32 0.00 109.84 1.101

IC CG CD2 CE2 HE21 1.537 110.81 0.00 109.79 1.101

IC HD21 CD2 CE2 HE21 1.101 109.57 59.91 109.79 1.101

IC HD22 CD2 CE2 HE21 1.101 109.32 -56.18 109.79 1.101

IC HZ2 CZ CE1 HE11 1.101 109.93 61.14 109.96 1.101

IC CE2 CZ CE1 HE11 1.532 111.19 0.00 109.96 1.101

IC HZ1 CZ CE1 HE11 1.101 109.83 -55.11 109.96 1.101

IC HZ2 CZ CE1 HE12 1.101 109.93 -55.03 109.82 1.101

IC CE2 CZ CE1 HE12 1.532 111.19 66.94 109.82 1.101

IC HZ1 CZ CE1 HE12 1.101 109.83 0.00 109.82 1.101

IC HZ2 CZ CE1 CD1 1.101 109.93 0.00 111.35 1.532

IC CE2 CZ CE1 CD1 1.532 111.19 -54.99 111.35 1.532

IC HZ1 CZ CE1 CD1 1.101 109.83 66.78 111.35 1.532

IC CG CD1 CE1 HE11 1.537 111.90 178.33 109.78 1.101

IC HD11 CD1 CE1 HE11 1.101 109.65 -59.76 109.78 1.101

IC HD12 CD1 CE1 HE11 1.101 109.31 56.33 109.78 1.101

IC CG CD1 CE1 HE12 1.537 111.90 -65.56 109.88 1.101

IC HD11 CD1 CE1 HE12 1.101 109.65 56.35 109.88 1.101

IC HD12 CD1 CE1 HE12 1.101 109.31 172.43 109.88 1.101

IC CG CD1 CE1 CZ 1.541 111.90 56.33 111.35 1.532

IC HD11 CD1 CE1 CZ 1.101 109.65 178.24 111.35 1.532

IC HD12 CD1 CE1 CZ 1.101 109.31 -65.67 111.35 1.532

PATCHING FIRS CHAN

**RESI GAB 0.00 ! L-Gamma amino butyric acid**

GROUP !

ATOM N NH1 -0.47 ! |

ATOM HN H 0.31 ! O=C

ATOM CG CXB2 -0.02 ! | HB1 HG1 HN

ATOM HG1 HXB2 0.09 ! | | | /

ATOM HG2 HXB2 0.09 ! HA1--CA--CB--CG--N

GROUP ! | | | \

ATOM CB CT2 -0.18 ! HA2 HB2 HG2

ATOM HB1 HA 0.09 !

ATOM HB2 HA 0.09 !

GROUP !

ATOM CA CXA2 -0.18 !

ATOM HA1 HXA2 0.09 !

ATOM HA2 HXA2 0.09 !

GROUP !

ATOM C C 0.51 !

ATOM O O -0.51 !

BOND CB CA N HN N CG

BOND C CA +N C CA HA1 CA HA2 CB HB1 HB2 CB

BOND CB CG CG HG1 CG HG2

DOUBLE O C

DONOR HN N

ACCEPTOR O C

IC -C N CB CA 1.3551 126.49 180.00 109.72 1.5100

IC -C N CG HG1 1.3551 126.49 180.00 114.44 1.1109

IC -C N CG HG2 1.3551 126.49 0.00 114.44 1.1109

IC CB CA C +N 1.5100 108.63 0.00 122.52 1.3558

IC CB CA C O 1.5100 108.63 0.00 122.52 1.3558

IC +N CA *C O 1.3558 116.84 180.00 122.52 1.2297

IC CA C +N +CA 1.5390 116.84 180.00 126.77 1.4613

IC C CA CB HB1 1.5390 108.63 -58.23 109.60 1.1109

IC HB1 CA *CB HB2 1.1109 109.60 -119.13 111.05 1.1119

IC HG1 CB *CG HG2 1.1109 109.60 -119.13 111.05 1.1119

IC +N C CA HA1 1.4613 116.84 120.45 106.39 1.0870

IC +N C CA HA2 1.4613 116.84 -125.45 106.39 1.0870

IC CB C *CA HA1 1.4592 114.44 120.05 106.39 1.0870

IC CB C *CA HA2 1.4592 114.44 -125.81 109.55 1.0870

IC -C CG *N HN 1.3476 123.81 180.00 114.54 0.9986

IC -C N CG CB 1.3476 123.81 180.00 113.64 1.5400

IC N CB *CG HG1 1.4501 106.52 122.27 112.34 1.1160

IC N CB *CG HG2 1.4501 106.52 -118.89 109.12 1.1160

IC CB C *CA HA1 1.5400 113.64 122.27 112.34 1.1150

IC CB C *CA HA2 1.5400 113.64 -118.89 112.34 1.1070

IC CG CA *CB HB1 1.5410 112.46 122.27 112.34 1.1090

IC CG CA *CB HB2 1.5410 112.46 -118.89 112.34 1.1100

IC O C CA CB 1.202 127.52 90.14 111.03 1.531

IC CG CB CA C 1.541 112.46 71.28 111.03 1.509

IC N CG CB CA 1.483 114.34 179.14 112.46 1.531

IC HB2 CB CA HA2 1.110 109.68 -176.66 111.07 1.107

IC HB2 CB CA HA1 1.110 109.68 67.72 109.42 1.115

IC HB2 CB CA C 1.110 109.68 -51.68 111.03 1.509

IC HG2 CG CB HB2 1.114 110.16 -179.53 110.17 1.110

IC N CG CB HB2 1.483 114.34 -58.17 110.17 1.110

IC HG1 CG CB HB2 1.116 110.14 63.32 110.17 1.110

**RESI BAL 0.00 !L- Betaalanine**

GROUP

ATOM N NH1 -0.47 !

ATOM HN H 0.31 ! -C

ATOM CB CXB2 -0.02 ! HA2 |

ATOM HB1 HXB2 0.09 ! | N--HN

ATOM HB2 HXB2 0.09 ! | /

GROUP ! HA--CA--CB-HB2

ATOM CA CXA2 -0.18 ! | \

ATOM HA1 HXA2 0.09 ! | HB1

ATOM HA2 HXA2 0.09 ! O=C--+N

GROUP !

ATOM C C 0.51

ATOM O O -0.51

BOND CB CA N HN N CB

BOND C CA +N C CA HA1 CA HA2 CB HB1 CB HB2

DOUBLE O C

DONOR HN N

ACCEPTOR O C

IC -C N CB CA 1.3551 126.49 180.00 109.72 1.5100

IC -C N CB HB1 1.3551 126.49 180.00 114.44 1.1109

IC -C N CB HB2 1.3551 126.49 0.00 114.44 1.1109

IC CB CA C +N 1.5100 108.63 180.00 116.84 1.3558

IC CB CA C O 1.5100 108.63 0.00 122.52 1.3558

IC +N CA *C O 1.3558 116.84 180.00 122.52 1.2297

IC CA C +N +CA 1.5390 116.84 180.00 126.77 1.4613

IC C CA CB HB1 1.5390 108.63 -58.23 109.60 1.1109

IC HB1 CA *CB HB2 1.1109 109.60 -119.13 111.05 1.1119

IC +N C CA HA1 1.4613 116.84 120.45 106.39 1.0870

IC +N C CA HA2 1.4613 116.84 -125.45 106.39 1.0870

IC CB C *CA HA1 1.4592 114.44 120.05 106.39 1.0870

IC CB C *CA HA2 1.4592 114.44 -125.81 109.55 1.0870

**RESI FPA 0.00 ! D- 4-Fluorophenyl alanine**

GROUP

ATOM N NH1 -0.47 ! | HD1 HE1

ATOM HN H 0.31 ! O=C | |

ATOM CA CT1 0.07 ! | HB1 CD1--CE1

ATOM HA HB 0.09 ! | | // \\

GROUP ! HN--N--CA--CB--CG CZ--F

ATOM CB CT2 -0.18 ! | | | \ __ /

ATOM HB1 HA 0.09 ! HA HB2 CD2--CE2

ATOM HB2 HA 0.09 ! | |

GROUP ! HD2 HE2

ATOM CG CA 0.00

GROUP

ATOM CD1 CA -0.115

ATOM HD1 HP 0.115

GROUP

ATOM CE1 CA -0.115

ATOM HE1 HP 0.115

GROUP

ATOM CZ CA 0.156

ATOM F FA -0.156

GROUP

ATOM CD2 CA -0.115

ATOM HD2 HP 0.115

GROUP

ATOM CE2 CA -0.115

ATOM HE2 HP 0.115

GROUP

ATOM C C 0.51

ATOM O O -0.51

BOND CB CA

BOND CG CB

BOND CD2 CG

BOND CE1 CD1

BOND CZ CE2

BOND N HN

BOND N CA

BOND C CA

BOND C +N

BOND CA HA

BOND CB HB1

BOND CB HB2

BOND CD1 HD1

BOND CD2 HD2

BOND CE1 HE1

BOND CE2 HE2

BOND CZ F

DOUBLE O C CD1 CG CZ CE1 CE2 CD2

IMPR N -C CA HN C CA +N O

IMPR CA N CB HA

DONOR HN N

ACCEPTOR O C

IC -C CA *N HN 1.3476 123.8900 180.0000 114.4700 0.9987

IC -C N CA C 1.3476 123.8900 180.0000 106.3800 1.5229

IC N CA C +N 1.4504 106.3800 180.0000 117.6500 1.3483

IC +N CA *C O 1.3483 117.6500 180.0000 120.4900 1.2287

IC CA C +N +CA 1.5229 117.6500 180.0000 124.1000 1.4523

IC N C *CA CB 1.4504 106.3800 -122.4900 112.4500 1.5594

IC N C *CA HA 1.4504 106.3800 115.6300 107.0500 1.0832

IC N CA CB CG 1.4504 111.6300 180.0000 112.7600 1.5109

IC CG CA *CB HB1 1.5109 112.7600 118.2700 109.1000 1.1119

IC CG CA *CB HB2 1.5109 112.7600 -123.8300 111.1100 1.1113

IC CA CB CG CD1 1.5594 112.7600 90.0000 120.3200 1.4059

IC CD1 CB *CG CD2 1.4059 120.3200 -177.9600 120.7600 1.4062

IC CB CG CD1 CE1 1.5109 120.3200 -177.3700 120.6300 1.4006

IC CE1 CG *CD1 HD1 1.4006 120.6300 179.7000 119.6500 1.0814

IC CB CG CD2 CE2 1.5109 120.7600 177.2000 120.6200 1.4002

IC CE2 CG *CD2 HD2 1.4002 120.6200 -178.6900 119.9900 1.0811

IC CG CD1 CE1 CZ 1.4059 120.6300 -0.1200 119.9300 1.4004

IC F CZ CE2 HE2 1.33 118.56 0.03 121.28 1.09

**RESI BPP +0.000 ! D-4-Benzoylphenylalanine**

GROUP

ATOM N NH1 -0.47

ATOM HN H 0.31

ATOM CA CT1 0.07

ATOM HA HB 0.09

GROUP

ATOM CB CT2 -0.18

ATOM HB1 HA 0.09

ATOM HB2 HA 0.09

GROUP

ATOM CG CA 0.00

GROUP

ATOM CD1 CA -0.115

ATOM HD1 HP 0.115

GROUP

ATOM CE1 CA -0.1055

ATOM HE1 HP 0.115

GROUP

ATOM CZ CA -0.087

GROUP

ATOM CD2 CA -0.115

ATOM HD2 HP 0.115

GROUP

ATOM CE2 CA -0.1055

ATOM HE2 HP 0.115

GROUP

ATOM CI CR 0.580

ATOM OI OR -0.425

GROUP

ATOM CJ CA -0.087

GROUP

ATOM CL1 CA -0.115

ATOM HL1 HP 0.115

GROUP

ATOM CK1 CA -0.115

ATOM HK1 HP 0.115

GROUP

ATOM CM CA -0.115

ATOM HM HP 0.115

GROUP

ATOM CK2 CA -0.115

ATOM HK2 HP 0.115

GROUP

ATOM CL2 CA -0.115

ATOM HL2 HP 0.115

GROUP

ATOM C C 0.51

ATOM O O -0.51

BOND CB CA CG CB CD2 CG CE1 CD1

BOND CZ CE2 N HN

BOND N CA C CA C +N CA HA

BOND CB HB1 CB HB2 CD1 HD1 CD2 HD2 CE1 HE1

DOUBLE O C CD1 CG CZ CE1 CE2 CD2

BOND CE2 HE2

BOND CZ CI CJ CI CJ CL2 CK1 CL1

BOND CM CK2

BOND CL1 HL1 CL2 HL2 CK1 HK1

DOUBLE OI CI CL1 CJ CM CK1 CK2 CL2

BOND CK2 HK2 CM HM

IMPR N -C CA HN C CA +N O

DONOR HN N

ACCEPTOR O C

ACCEPTOR OI CI

IC -C CA *N HN 1.3476 123.81 180.00 114.54 0.9986

IC CA C +N +CA 1.5232 117.33 180.00 124.31 1.4513

IC -C N CA C 1.3476 123.81 180.00 106.52 1.5232

IC N CA C +N 1.4501 106.52 180.00 117.33 1.3484

IC +N CA *C O 1.3484 117.33 180.00 120.67 1.2287

IC N C *CA CB 1.4501 106.52 -122.27 112.34 1.5606

IC N C *CA HA 1.4501 106.52 116.04 107.15 1.0833

IC CG CA *CB HB1 1.5113 112.94 118.89 109.12 1.1119

IC CG CA *CB HB2 1.5113 112.94 -123.36 110.70 1.1115

IC CD1 CB *CG CD2 1.4064 120.49 -176.46 120.46 1.4068

IC CE1 CG *CD1 HD1 1.4026 120.40 178.94 119.80 1.0814

IC CE2 CG *CD2 HD2 1.4022 120.56 -177.57 119.98 1.0813

IC CZ CD1 *CE1 HE1 1.3978 120.09 179.64 120.58 1.0799

IC CE1 CE2 *CZ CI 1.3978 120.05 -178.98 120.25 1.4970

IC CJ CZ *CI OI 1.4910 117.33 180.00 120.67 1.1213

IC CK1 CJ *CL1 HL1 1.4026 120.40 178.94 119.80 1.0814

IC CK2 CJ *CL2 HL2 1.4022 120.56 -177.57 119.98 1.0813

IC CM CL1 *CK1 HK1 1.3978 120.09 179.64 120.58 1.0799

IC CM CL2 *CK2 HK2 1.3979 119.92 -178.69 119.76 1.0811

IC CK1 CK2 *CM HM 1.3978 120.05 -178.98 120.25 1.0807

IC CB CA N HN 1.546 115.71 -66.50 110.76 1.018

IC C CA N HN 1.530 106.15 173.71 110.76 1.018

IC HA CA N HN 1.128 107.98 56.82 110.76 1.018

IC O C CA CB 1.205 130.22 -65.88 108.02 1.546

IC O C CA N 1.205 130.22 58.80 106.15 1.480

IC O C CA HA 1.205 130.22 174.93 109.12 1.128

IC HB2 CB CA C 1.112 109.82 -56.28 108.02 1.530

IC CG CB CA C 1.502 110.82 -178.13 108.02 1.530

IC HB1 CB CA C 1.111 110.18 58.45 108.02 1.530

IC HB2 CB CA N 1.112 109.82 -175.04 115.71 1.480

IC CG CB CA N 1.502 110.82 63.11 115.71 1.480

IC HB1 CB CA N 1.111 110.18 -60.31 115.71 1.480

IC HB2 CB CA HA 1.112 109.82 62.55 109.69 1.128

IC CG CB CA HA 1.502 110.82 -59.31 109.69 1.128

IC HB1 CB CA HA 1.111 110.18 177.27 109.69 1.128

IC CD2 CG CB HB2 1.403 120.17 137.40 110.09 1.112

IC CD1 CG CB HB2 1.404 120.06 -43.34 110.09 1.112

IC CD2 CG CB CA 1.403 120.17 -100.91 110.82 1.546

IC CD1 CG CB CA 1.404 120.06 78.35 110.82 1.546

IC CD2 CG CB HB1 1.403 120.17 21.96 111.14 1.111

IC CD1 CG CB HB1 1.404 120.06 -158.78 111.14 1.111

IC HD1 CD1 CG CB 1.089 120.09 0.32 120.06 1.502

IC CE1 CD1 CG CB 1.395 120.13 -179.72 120.06 1.502

IC HD1 CD1 CG CD2 1.089 120.09 179.58 119.76 1.403

IC CE1 CD1 CG CD2 1.395 120.13 -0.46 119.76 1.403

IC HD2 CD2 CG CB 1.089 120.31 -0.82 120.17 1.502

IC CE2 CD2 CG CB 1.396 120.01 179.34 120.17 1.502

IC HD2 CD2 CG CD1 1.089 120.31 179.92 119.76 1.404

IC CE2 CD2 CG CD1 1.396 120.01 0.08 119.76 1.404

IC CD2 CE2 CZ CE1 1.396 120.22 -0.94 119.75 1.402

IC HE2 CE2 CZ CE1 1.094 119.35 178.68 119.75 1.402

IC CD2 CE2 CZ CI 1.396 120.22 -178.98 118.61 1.497

IC HE2 CE2 CZ CI 1.094 119.35 0.65 118.61 1.497

IC HE1 CE1 CZ CE2 1.092 120.02 -179.04 119.75 1.403

IC CD1 CE1 CZ CE2 1.395 120.12 0.56 119.75 1.403

IC HE1 CE1 CZ CI 1.092 120.02 -1.07 121.61 1.497

IC CD1 CE1 CZ CI 1.395 120.12 178.53 121.61 1.497

IC CJ CI CZ CE1 1.496 117.59 34.09 121.61 1.402

IC OI CI CZ CE1 1.213 121.01 -145.82 121.61 1.402

IC CJ CI CZ CE2 1.496 117.59 -147.92 118.61 1.403

IC OI CI CZ CE2 1.213 121.01 32.17 118.61 1.403

IC CZ CE1 CD1 HD1 1.402 120.12 -179.90 119.78 1.089

IC HE1 CE1 CD1 HD1 1.092 119.85 -0.30 119.78 1.089

IC CZ CE1 CD1 CG 1.402 120.12 0.14 120.13 1.404

IC HE1 CE1 CD1 CG 1.092 119.85 179.74 120.13 1.404

IC CZ CE2 CD2 HD2 1.403 120.22 -179.22 119.68 1.089

IC HE2 CE2 CD2 HD2 1.094 120.43 1.16 119.68 1.089

IC CZ CE2 CD2 CG 1.403 120.22 0.62 120.01 1.403

IC HE2 CE2 CD2 CG 1.094 120.43 -179.00 120.01 1.403

IC CL2 CJ CI CZ 1.402 121.27 39.80 117.59 1.497

IC CL1 CJ CI CZ 1.403 118.67 -142.22 117.59 1.497

IC CL2 CJ CI OI 1.402 121.27 -140.29 121.40 1.213

IC CL1 CJ CI OI 1.403 118.67 37.68 121.40 1.213

IC CK1 CL1 CJ CL2 1.397 119.91 -1.24 120.03 1.402

IC HL1 CL1 CJ CL2 1.093 119.58 178.34 120.03 1.402

IC CK1 CL1 CJ CI 1.397 119.91 -179.24 118.67 1.496

IC HL1 CL1 CJ CI 1.093 119.58 0.34 118.67 1.496

IC HL2 CL2 CJ CL1 1.091 120.11 -178.79 120.03 1.403

IC CK2 CL2 CJ CL1 1.397 119.85 0.76 120.03 1.403

IC HL2 CL2 CJ CI 1.091 120.11 -0.84 121.27 1.496

IC CK2 CL2 CJ CI 1.397 119.85 178.71 121.27 1.496

IC HK2 CK2 CM HM 1.089 120.03 -0.39 119.91 1.090

IC CL2 CK2 CM HM 1.397 120.08 179.78 119.91 1.090

IC HK2 CK2 CM CK1 1.089 120.03 179.35 120.13 1.399

IC CL2 CK2 CM CK1 1.397 120.08 -0.48 120.13 1.399

IC CL1 CK1 CM CK2 1.397 119.99 0.00 120.13 1.399

IC HK1 CK1 CM CK2 1.089 120.10 -179.51 120.13 1.399

IC CL1 CK1 CM HM 1.397 119.99 179.74 119.96 1.090

IC HK1 CK1 CM HM 1.089 120.10 0.22 119.96 1.090

IC HK1 CK1 CL1 CJ 1.089 119.92 -179.62 119.91 1.403

IC CM CK1 CL1 CJ 1.399 119.99 0.86 119.91 1.403

IC HK1 CK1 CL1 HL1 1.089 119.92 0.80 120.51 1.093

IC CM CK1 CL1 HL1 1.399 119.99 -178.72 120.51 1.093

IC HK2 CK2 CL2 HL2 1.089 119.89 -0.18 120.04 1.091

IC CM CK2 CL2 HL2 1.399 120.08 179.65 120.04 1.091

IC HK2 CK2 CL2 CJ 1.089 119.89 -179.73 119.85 1.402

IC CM CK2 CL2 CJ 1.399 120.08 0.10 119.85 1.402

**RESI TFP 0.00 ! L, Trans-4-fluoroproline**

GROUP ! HD1 HD2

ATOM N N -0.29 ! | \ /

ATOM CD CP3 0.00 ! N---CD HG

ATOM HD1 HA 0.09 ! | \ /

ATOM HD2 HA 0.09 ! | CG

ATOM CA CP1 0.02 ! | / \

ATOM HA HB 0.09 ! HA-CA--CB F

GROUP ! | / \

ATOM CB CP2 -0.18 ! | HB1 HB2

ATOM HB1 HA 0.09 ! O=C

ATOM HB2 HA 0.09 ! |

GROUP

ATOM CG CP2 0.11

ATOM HG HA 0.11

ATOM F FN1 -0.22

GROUP

ATOM C C 0.51

ATOM O O -0.51

BOND C CA C +N

BOND N CA CA CB CB CG CG CD N CD

BOND HA CA HG CG F CG HD1 CD HD2 CD HB1 CB HB2 CB

DOUBLE O C

IMPR N -C CA CD

IMPR C CA +N O

ACCEPTOR O C

IC -C CA *N CD 1.3366 122.94 178.51 112.75 1.4624

IC -C N CA C 1.3366 122.94 -76.12 110.86 1.5399

IC N CA C +N 1.4585 110.86 180.00 114.75 1.3569

IC +N CA *C O 1.3569 114.75 177.15 120.46 1.2316

IC CA C +N +CA 1.5399 116.12 180.00 124.89 1.4517

IC N C *CA CB 1.4585 110.86 113.74 111.74 1.5399

IC N C *CA HA 1.4585 110.86 -122.40 109.09 1.0837

IC N CA CB CG 1.4585 102.56 31.61 104.39 1.5322

IC CA CB CG CD 1.5399 104.39 -34.59 103.21 1.5317

IC N CA CB HB1 1.4585 102.56 -84.94 109.02 1.1131

IC N CA CB HB2 1.4585 102.56 153.93 112.74 1.1088

IC CA CB CG HG 1.5399 104.39 -156.72 112.95 1.1077

IC CA CB CG F 1.5399 104.39 81.26 108.58 1.3810

IC CB CG CD HD1 1.5322 103.21 -93.55 110.03 1.1137

IC CB CG CD HD2 1.5322 103.21 144.52 110.00 1.1144

PATCHING FIRS TFPN

**RESI NPA +0.000 ! D, 1-Naphthyl-alanine**

GROUP

ATOM N NH1 -0.47 ! | HD HE

ATOM HN H 0.31 ! O=C | |

ATOM CA CT1 0.07 ! | HB1 CD1--CE1

ATOM HA HB 0.09 ! | | // \\

GROUP HN--N--CA--CB--CG CZ--HZ

ATOM CB CT2 -0.18 ! | | | \ __ /

ATOM HB1 HA 0.09 ! HA HB2 CD2--CE2

ATOM HB2 HA 0.09 ! / \

GROUP HV--CV CY--HY

ATOM CG CA 0.00 ! \\ //

GROUP CW ---CX

ATOM CD1 CA -0.115! | |

ATOM HD HP 0.115! HW HX

GROUP

ATOM CE1 CA -0.115

ATOM HE HP 0.115

GROUP

ATOM CZ CA -0.115

ATOM HZ HP 0.115

GROUP

ATOM CE2 CAN 0.00

GROUP

ATOM CD2 CAN 0.00

GROUP

ATOM CV CA -0.115

ATOM HV HP 0.115

GROUP

ATOM CW CA -0.115

ATOM HW HP 0.115

GROUP

ATOM CX CA -0.115

ATOM HX HP 0.115

GROUP

ATOM CY CA -0.115

ATOM HY HP 0.115

GROUP

ATOM C C 0.51

ATOM O O -0.51

BOND CA N N HN C CA

BOND CA HA CB CA

BOND CB HB1 CB HB2 CG CB

BOND CE1 CD1 CD1 HD CD2 CG

BOND CE2 CZ CZ HZ

BOND CV CD2 CE1 HE CY CE2

BOND CV HV CX CW CW HW

BOND CX HX CY HY

DOUBLE O C CG CD1 CE1 CZ CE2 CD2 CV CW CX CY

IMPR N -C CA HN C CA +N O

IMPR CE1 CD1 CZ HE

IMPR CD1 CE1 CG HD

IMPR CV CW CD2 HV

IMPR CW CX CV HW

IMPR CY CE2 CW HY

DONOR HN N

ACCEPTOR O C

IC C CA N HN 1.541 112.31 -72.85 111.20 1.020

IC HA CA N HN 1.129 106.68 170.21 111.20 1.020

IC CB CA N HN 1.542 110.80 52.10 111.20 1.020

IC O C CA N 1.202 126.65 -2.40 112.31 1.478

IC O C CA HA 1.202 126.65 114.34 107.01 1.129

IC O C CA CB 1.202 126.65 -127.16 111.14 1.542

IC CG CB CA N 1.508 113.74 -66.60 110.80 1.478

IC HB1 CB CA N 1.117 107.79 55.77 110.80 1.478

IC HB2 CB CA N 1.108 110.00 169.42 110.80 1.478

IC CG CB CA C 1.508 113.74 59.00 111.14 1.541

IC HB1 CB CA C 1.117 107.79 -178.63 111.14 1.541

IC HB2 CB CA C 1.108 110.00 -64.98 111.14 1.541

IC CG CB CA HA 1.508 113.74 176.50 108.67 1.129

IC HB1 CB CA HA 1.117 107.79 -61.13 108.67 1.129

IC HB2 CB CA HA 1.108 110.00 52.52 108.67 1.129

IC CD2 CG CB CA 1.439 120.50 98.00 113.74 1.542

IC CD1 CG CB CA 1.378 119.59 -82.76 113.74 1.542

IC CD2 CG CB HB1 1.439 120.50 -23.09 110.10 1.117

IC CD1 CG CB HB1 1.378 119.59 156.15 110.10 1.117

IC CD2 CG CB HB2 1.439 120.50 -138.05 110.07 1.108

IC CD1 CG CB HB2 1.378 119.59 41.18 110.07 1.108

IC CD2 CG CD1 CE1 1.439 119.90 0.08 120.89 1.423

IC CB CG CD1 CE1 1.508 119.59 -179.16 120.89 1.423

IC CD2 CG CD1 HD 1.439 119.90 -179.13 121.21 1.091

IC CB CG CD1 HD 1.508 119.59 1.63 121.21 1.091

IC CZ CE1 CD1 HD 1.371 120.36 179.47 117.90 1.091

IC HE CE1 CD1 HD 1.090 118.56 -0.35 117.90 1.091

IC CZ CE1 CD1 CG 1.371 120.36 0.24 120.89 1.378

IC HE CE1 CD1 CG 1.090 118.56 -179.59 120.89 1.378

IC CV CD2 CG CB 1.433 122.70 -1.16 120.50 1.508

IC CE2 CD2 CG CB 1.420 118.84 178.89 120.50 1.508

IC CV CD2 CG CD1 1.433 122.70 179.60 119.90 1.378

IC CE2 CD2 CG CD1 1.420 118.84 -0.35 119.90 1.378

IC HE CE1 CZ CE2 1.090 121.07 179.55 120.25 1.430

IC CD1 CE1 CZ CE2 1.423 120.36 -0.27 120.25 1.430

IC HE CE1 CZ HZ 1.090 121.07 -0.25 120.93 1.089

IC CD1 CE1 CZ HZ 1.423 120.36 179.93 120.93 1.089

IC CY CE2 CZ HZ 1.433 120.76 -0.07 118.82 1.089

IC CD2 CE2 CZ HZ 1.420 119.76 179.80 118.82 1.089

IC CY CE2 CZ CE1 1.433 120.76 -179.87 120.25 1.371

IC CD2 CE2 CZ CE1 1.420 119.76 -0.01 120.25 1.371

IC CY CE2 CD2 CV 1.433 119.48 0.23 118.46 1.433

IC CZ CE2 CD2 CV 1.430 119.76 -179.64 118.46 1.433

IC CY CE2 CD2 CG 1.433 119.48 -179.82 118.84 1.439

IC CZ CE2 CD2 CG 1.430 119.76 0.31 118.84 1.439

IC CW CV CD2 CG 1.371 120.96 179.79 122.70 1.439

IC HV CV CD2 CG 1.093 118.56 -0.69 122.70 1.439

IC CW CV CD2 CE2 1.371 120.96 -0.26 118.46 1.420

IC HV CV CD2 CE2 1.093 118.56 179.26 118.46 1.420

IC HY CY CE2 CZ 1.090 118.52 -0.11 120.76 1.430

IC CX CY CE2 CZ 1.370 120.57 179.80 120.76 1.430

IC HY CY CE2 CD2 1.090 118.52 -179.98 119.48 1.420

IC CX CY CE2 CD2 1.370 120.57 -0.07 119.48 1.420

IC HW CW CV CD2 1.089 120.85 179.84 120.96 1.433

IC CX CW CV CD2 1.426 120.38 0.12 120.96 1.433

IC HW CW CV HV 1.089 120.85 0.34 120.48 1.093

IC CX CW CV HV 1.426 120.38 -179.38 120.48 1.093

IC CY CX CW CV 1.370 120.15 0.05 120.38 1.371

IC HX CX CW CV 1.089 118.77 179.98 120.38 1.371

IC CY CX CW HW 1.370 120.15 -179.68 118.78 1.089

IC HX CX CW HW 1.089 118.77 0.25 118.78 1.089

IC HY CY CX HX 1.090 120.90 -0.09 121.07 1.089

IC CE2 CY CX HX 1.433 120.57 179.99 121.07 1.089

IC HY CY CX CW 1.090 120.90 179.84 120.15 1.426

IC CE2 CY CX CW 1.433 120.57 -0.07 120.15 1.426

IC -C CA *N HN 1.3476 123.8900 180.0000 114.4700 0.9987

IC -C N CA C 1.3476 123.8900 180.0000 106.3800 1.5229

IC N CA C +N 1.4504 106.3800 180.0000 117.6500 1.3483

IC +N CA *C O 1.3483 117.6500 180.0000 120.4900 1.2287

IC CA C +N +CA 1.5229 117.6500 180.0000 124.1000 1.4523

IC N C *CA CB 1.4504 106.3800 -122.4900 112.4500 1.5594

IC N C *CA HA 1.4504 106.3800 115.6300 107.0500 1.0832

IC CG CA *CB HB1 1.5109 112.7600 118.2700 109.1000 1.1119

IC CG CA *CB HB2 1.5109 112.7600 -123.8300 111.1100 1.1113

IC CD1 CB *CG CD2 1.4059 120.3200 -177.9600 120.7600 1.4062

IC CE1 CG *CD1 HD 1.4006 120.6300 179.7000 119.6500 1.0814

IC CE2 CG *CD2 CV 1.4002 120.6200 -178.6900 119.9900 1.4330

IC CZ CD1 *CE1 HE 1.4004 119.9300 -179.6900 120.0100 1.0808

IC CZ CD2 *CE2 CY 1.4000 119.9600 -179.9300 119.8700 1.4330

IC CE1 CE2 *CZ HZ 1.4004 119.9800 179.5100 119.9700 1.1090

IC CX CV *CW HW 1.4006 120.6300 179.7000 119.6500 1.0814

IC CY CW *CX HX 1.4004 119.9300 -179.6900 120.0100 1.0808

IC CE2 CX *CY HY 1.4004 119.9800 179.5100 119.9700 1.1090

IC CW CD2 *CV HV 1.4004 119.9800 179.5100 119.9700 1.1090

**RESI PGL +0.000 ! D-Phenylglycine**

GROUP

ATOM N NH1 -0.47 ! | HG1 HD1

ATOM HN H +0.31 ! O=C | |

ATOM CA CT1 +0.07 ! | CG1--CD1

ATOM HA HB +0.09 ! | // \\

GROUP ! HN--N--CA--CB CE--HE

ATOM CB CA 0.00 ! | | \ __ /

GROUP ! HA CG2--CD2

ATOM CG1 CA -0.115 ! | |

ATOM HG1 HP 0.115 ! HG2 HD2

GROUP

ATOM CG2 CA -0.115

ATOM HG2 HP 0.115

GROUP

ATOM CD1 CA -0.115

ATOM HD1 HP 0.115

GROUP

ATOM CD2 CA -0.115

ATOM HD2 HP 0.115

GROUP

ATOM CE CA -0.115

ATOM HE HP 0.115

GROUP

ATOM C C 0.510

ATOM O O -0.510

BOND CA N

BOND HN N

BOND C CA

BOND HA CA

BOND CB CA

BOND CG2 CB

BOND CD2 CE

BOND HE CE

BOND CD1 CG1

BOND HG1 CG1

BOND HG2 CG2

BOND HD2 CD2

BOND HD1 CD1

DOUBLE O C

DOUBLE CG1 CB

DOUBLE CE CD1

DOUBLE CD2 CG2

IMPR N -C CA HN C CA +N O

DONOR HN N

ACCEPTOR O C

IC -C CA *N HN 1.3476 123.8900 180.0000 114.4700 0.9987

IC -C N CA C 1.3476 123.8900 180.0000 106.3800 1.5229

IC N CA C +N 1.4504 106.3800 180.0000 117.6500 1.3483

IC +N CA *C O 1.3483 117.6500 180.0000 120.4900 1.2287

IC CA C +N +CA 1.5229 117.6500 180.0000 124.1000 1.4523

IC N C *CA CB 1.4504 106.3800 -122.4900 112.4500 1.5594

IC N C *CA HA 1.4504 106.3800 115.6300 107.0500 1.0832

IC CG1 CA *CB CG2 1.4059 120.3200 -177.9600 120.7600 1.4062

IC CD1 CB *CG1 HG1 1.4006 120.6300 179.7000 119.6500 1.0814

IC CD2 CB *CG2 HG2 1.4002 120.6200 -178.6900 119.9900 1.0811

IC CE CG1 *CD1 HD1 1.4004 119.9300 -179.6900 120.0100 1.0808

IC CE CG2 *CD2 HD2 1.4000 119.9600 -179.9300 119.8700 1.0811

IC CD1 CD2 *CE HE 1.4004 119.9800 179.5100 119.9700 1.0807 !taken from PHE

IC CB CA N HN 1.513 112.53 -41.14 110.54 1.020

IC C CA N HN 1.540 107.61 84.34 110.54 1.020

IC HA CA N HN 1.130 108.60 -162.39 110.54 1.020

IC O C CA CB 1.204 126.32 -31.57 113.27 1.513

IC O C CA N 1.204 126.32 -156.61 107.61 1.491

IC O C CA HA 1.204 126.32 87.78 105.09 1.130

IC CG1 CB CA C 1.402 121.36 -35.94 113.27 1.540

IC CG2 CB CA C 1.404 118.82 146.22 113.27 1.540

IC CG1 CB CA N 1.402 121.36 86.40 112.53 1.491

IC CG2 CB CA N 1.404 118.82 -91.44 112.53 1.491

IC CG1 CB CA HA 1.402 121.36 -152.80 109.39 1.130

IC CG2 CB CA HA 1.404 118.82 29.36 109.39 1.130

IC CD1 CG1 CB CG2 1.399 119.94 0.06 119.79 1.404

IC HG1 CG1 CB CG2 1.091 120.39 -178.16 119.79 1.404

IC CD1 CG1 CB CA 1.399 119.94 -177.76 121.36 1.513

IC HG1 CG1 CB CA 1.091 120.39 4.02 121.36 1.513

IC CD2 CG2 CB CG1 1.396 119.99 -0.19 119.79 1.402

IC HG2 CG2 CB CG1 1.088 120.33 179.34 119.79 1.402

IC CD2 CG2 CB CA 1.396 119.99 177.68 118.82 1.513

IC HG2 CG2 CB CA 1.088 120.33 -2.79 118.82 1.513

IC CG2 CD2 CE HE 1.396 120.21 179.71 120.04 1.089

IC HD2 CD2 CE HE 1.090 119.93 -0.01 120.04 1.089

IC CG2 CD2 CE CD1 1.396 120.21 0.15 119.84 1.398

IC HD2 CD2 CE CD1 1.090 119.93 -179.58 119.84 1.398

IC HD1 CD1 CE CD2 1.090 120.04 179.16 119.84 1.399

IC CG1 CD1 CE CD2 1.399 120.22 -0.28 119.84 1.399

IC HD1 CD1 CE HE 1.090 120.04 -0.40 120.13 1.089

IC CG1 CD1 CE HE 1.399 120.22 -179.84 120.13 1.089

IC HD1 CD1 CG1 CB 1.090 119.73 -179.27 119.94 1.402

IC CE CD1 CG1 CB 1.398 120.22 0.17 119.94 1.402

IC HD1 CD1 CG1 HG1 1.090 119.73 -1.04 119.64 1.091

IC CE CD1 CG1 HG1 1.398 120.22 178.41 119.64 1.091

IC CE CD2 CG2 HG2 1.399 120.21 -179.45 119.68 1.088

IC HD2 CD2 CG2 HG2 1.090 119.86 0.28 119.68 1.088

IC CE CD2 CG2 CB 1.399 120.21 0.09 119.99 1.404

IC HD2 CD2 CG2 CB 1.090 119.86 179.81 119.99 1.404

**RESI HCT +0.00 ! D-Homocitrulline**

GROUP !

ATOM N NH1 -0.47 ! |

ATOM HN H 0.31 ! HN-N

ATOM CA CT1 0.07 ! | HB1 HG1 HD1 HE1 HZ OR

ATOM HA HB 0.09 ! | | | | | | //

GROUP ! HA-CA--CB--CG--CD--CE--NZ--CQ

ATOM CB CT2 -0.18 ! | | | | | \

ATOM HB1 HA 0.09 ! | HB2 HG2 HD2 HE2 NR-HR1

ATOM HB2 HA 0.09 ! O=C |

GROUP ! | HR2

ATOM CG CT2 -0.18 !

ATOM HG1 HA 0.09

ATOM HG2 HA 0.09

GROUP

ATOM CD CT2 -0.18

ATOM HD2 HA 0.09

ATOM HD1 HA 0.09

GROUP

ATOM CE CT2 0.20

ATOM HE1 HA 0.09

ATOM HE2 HA 0.09

ATOM NZ NC2 -0.70

ATOM HZ HC 0.44

ATOM CQ CQ 0.422

ATOM OR OQ -0.662

ATOM NR NC2 -0.80

ATOM HR1 HC 0.46

ATOM HR2 HC 0.46

GROUP

ATOM C C 0.51

ATOM O O -0.51

BOND CA N

BOND HN N

BOND C CA

BOND HA CA

BOND CB CA

BOND

BOND HB1 CB

BOND HB2 CB

BOND CG CB

BOND HG1 CG

BOND HG2 CG

BOND CD CG

BOND HD1 CD

BOND CE CD

BOND HD2 CD

BOND HE1 CE

BOND HE2 CE

BOND NZ CE

BOND HZ NZ

BOND CQ NZ

BOND NR CQ

BOND HR1 NR

BOND HR2 NR

DOUBLE O C

DOUBLE OR CQ

IMPR N -C CA HN

IMPR C CA +N O

IMPR CQ O NR NZ

DONOR HN N

DONOR HZ NZ

DONOR HR1 NR

DONOR HR2 NR

ACCEPTOR O C

ACCEPTOR OR CQ

IC -C CA *N HN 1.3476 123.8900 180.0000 114.4700 0.9987

IC -C N CA C 1.3476 123.8900 180.0000 106.3800 1.5229

IC N CA C +N 1.4504 106.3800 180.0000 117.6500 1.3483

IC +N CA *C O 1.3483 117.6500 180.0000 120.4900 1.2287

IC CA C +N +CA 1.5229 117.6500 180.0000 124.1000 1.4523

IC N C *CA CB 1.4504 106.3800 -122.4900 112.4500 1.5594

IC N C *CA HA 1.4504 106.3800 115.6300 107.0500 1.0832

IC CG CA *CB HB1 1.5475 115.9500 120.0500 106.4000 1.1163

IC CG CA *CB HB2 1.5475 115.9500 -125.8100 109.5500 1.1124

IC CD CB *CG HG1 1.5384 114.0100 125.2000 108.5500 1.1121

IC CD CB *CG HG2 1.5384 114.0100 -120.3000 108.9600 1.1143

IC CE CG *CD HD1 1.5384 114.0100 125.2000 108.5500 1.1121

IC CE CG *CD HD2 1.5384 114.0100 -120.3000 108.9600 1.1143

IC NZ CD *CE HE1 1.5034 107.0900 120.6900 109.4100 1.1143

IC NZ CD *CE HE2 1.5034 107.0900 -119.0400 111.5200 1.1150

IC CQ CE *NZ HZ 1.3401 123.0500 180.0000 113.1400 1.0065

IC NR NZ *CQ O 1.3483 118.7500 180.0000 120.4900 1.2287

IC HR1 CQ *NR HR2 0.9903 120.6100 171.1900 116.2900 1.0023

IC HA CA N HN 1.1280 108.4800 40.1000 110.9400 1.0170

IC C CA N HN 1.5390 107.7900 154.2800 110.9400 1.0170

IC CB CA N HN 1.5390 115.0900 -81.4000 110.9400 1.0170

IC O C CA HA 1.2060 125.4100 -85.6900 105.8500 1.1280

IC O C CA N 1.2060 125.4100 158.3900 107.7900 1.4860

IC O C CA CB 1.2060 125.4100 31.5900 110.8900 1.5390

IC HB2 CB CA HA 1.1120 108.5400 51.5800 108.3300 1.1280

IC HB1 CB CA HA 1.1070 109.4000 -63.4100 108.3300 1.1280

IC CG CB CA HA 1.5340 113.4200 172.8500 108.3300 1.1280

IC HB2 CB CA C 1.1120 108.5400 -64.1700 110.8900 1.5390

IC HB1 CB CA C 1.1070 109.4000 -179.1600 110.8900 1.5390

IC CG CB CA C 1.5340 113.4200 57.1100 110.8900 1.5390

IC HB2 CB CA N 1.1120 108.5400 173.1600 115.0900 1.4860

IC HB1 CB CA N 1.1070 109.4000 58.1700 115.0900 1.4860

IC CG CB CA N 1.5340 113.4200 -65.5600 115.0900 1.4860

IC HG1 CG CB HB2 1.1130 109.1800 57.1900 108.9900 1.1120

IC HG2 CG CB HB2 1.1070 110.6400 173.5200 108.9900 1.1120

IC CD CG CB HB2 1.5330 111.1000 -64.0500 108.9900 1.1120

IC HG1 CG CB HB1 1.1130 109.1800 172.9800 110.4100 1.1070

IC HG2 CG CB HB1 1.1070 110.6400 -70.6900 110.4100 1.1070

IC CD CG CB HB1 1.5330 111.1000 51.7400 110.4100 1.1070

IC HG1 CG CB CA 1.1130 109.1800 -63.8400 113.4200 1.5390

IC HG2 CG CB CA 1.1070 110.6400 52.5000 113.4200 1.5390

IC CD CG CB CA 1.5330 111.1000 174.9300 113.4200 1.5390

IC HD2 CD CG HG1 1.1090 110.2800 177.5600 109.7900 1.1130

IC CE CD CG HG1 1.5390 110.6600 55.7500 109.7900 1.1130

IC HD1 CD CG HG1 1.1090 109.9300 -66.0500 109.7900 1.1130

IC HD2 CD CG HG2 1.1090 110.2800 61.2500 109.9600 1.1070

IC CE CD CG HG2 1.5390 110.6600 -60.5500 109.9600 1.1070

IC HD1 CD CG HG2 1.1090 109.9300 177.6500 109.9600 1.1070

IC HD2 CD CG CB 1.1090 110.2800 -61.5700 111.1000 1.5340

IC CE CD CG CB 1.5390 110.6600 176.6200 111.1000 1.5340

IC HD1 CD CG CB 1.1090 109.9300 54.8300 111.1000 1.5340

IC HE2 CE CD HD2 1.1150 109.6200 -65.7300 109.9000 1.1090

IC HE1 CE CD HD2 1.1150 110.2000 176.2000 109.9000 1.1090

IC NZ CE CD HD2 1.4800 110.0100 55.4600 109.9000 1.1090

IC HE2 CE CD CG 1.1150 109.6200 56.3000 110.6600 1.5330

IC HE1 CE CD CG 1.1150 110.2000 -61.7700 110.6600 1.5330

IC NZ CE CD CG 1.4800 110.0100 177.4900 110.6600 1.5330

IC HE2 CE CD HD1 1.1150 109.6200 178.0200 110.0800 1.1090

IC HE1 CE CD HD1 1.1150 110.2000 59.9500 110.0800 1.1090

IC NZ CE CD HD1 1.4800 110.0100 -60.8000 110.0800 1.1090

IC HZ NZ CE CD 1.0190 117.6800 28.5100 110.0100 1.5390

IC CQ NZ CE CD 1.4000 125.7200 -150.6800 110.0100 1.5390

IC HZ NZ CE HE2 1.0190 117.6800 149.4200 110.0800 1.1150

IC CQ NZ CE HE2 1.4000 125.7200 -29.7700 110.0800 1.1150

IC HZ NZ CE HE1 1.0190 117.6800 -92.6900 109.4300 1.1150

IC CQ NZ CE HE1 1.4000 125.7200 88.1100 109.4300 1.1150

IC OR CQ NZ HZ 1.2240 120.0500 -4.3600 116.6000 1.0190

IC NR CQ NZ HZ 1.4180 118.7500 178.3400 116.6000 1.0190

IC OR CQ NZ CE 1.2240 120.0500 174.8400 125.7200 1.4800

IC NR CQ NZ CE 1.4180 118.7500 -2.4500 125.7200 1.4800

IC HR1 NR CQ OR 1.0070 120.0800 150.5300 121.1400 1.2240

IC HR2 NR CQ OR 1.0150 114.5900 10.0100 121.1400 1.2240

IC HR1 NR CQ NZ 1.0070 120.0800 -32.2000 118.7500 1.4000

IC HR2 NR CQ NZ 1.0150 114.5900 -172.730 118.7500 1.4000

**RESI LBH +1.000 ! L-Betahomoarginie**

GROUP !

ATOM N NH1 -0.47 ! | HR11

ATOM HN H 0.31 ! HN-N |

ATOM CB CT1 0.07 ! HA1 | HG1 HD1 HE1 NR1-HR12

ATOM HB HA 0.09 ! | | | | | //(+)

GROUP ! HA2-CA--CB--CG--CD--CE--NZ--CQ

ATOM CA CT2 -0.18 ! | | | | | | \

ATOM HA HA 0.09 ! | HB HG2 HD2 HE2 HZ NR2-HR22

ATOM HA2 HA 0.09 ! O=C |

GROUP ! | HR21

ATOM CG CT2 -0.18

ATOM HG1 HA 0.09

ATOM HG2 HA 0.09

GROUP

ATOM CD CT2 -0.18

ATOM HD1 HA 0.09

ATOM HD2 HA 0.09

GROUP

ATOM CE CT2 0.20

ATOM HE1 HA 0.09

ATOM HE2 HA 0.09

ATOM NZ NC2 -0.70

ATOM HZ HC 0.44

ATOM CQ C 0.64

ATOM NR1 NC2 -0.80

ATOM HR11 HC 0.46

ATOM HR12 HC 0.46

ATOM NR2 NC2 -0.80

ATOM HR21 HC 0.46

ATOM HR22 HC 0.46

GROUP

ATOM C C 0.51

ATOM O O 0.51

BOND CB N

BOND HN N

BOND HB CB

BOND CG CB

BOND CA CB

BOND HG1 CG

BOND HG2 CG

BOND CD CG

BOND HD1 CD

BOND HD2 CD

BOND CE CD

BOND HE1 CE

BOND HE2 CE

BOND NZ CE

BOND C CA

BOND HA CA

BOND HA2 CA

BOND CQ NZ

BOND HZ NZ

BOND NR2 CQ

BOND HR11 NR1

BOND HR12 NR1

BOND HR21 NR2

BOND HR22 NR2

DOUBLE O C CQ NR1

IMPR N -C CB HN C CA +N O

!IMPR CQ NR1 NR2 NZ

DONOR HN N

DONOR HZ NZ

DONOR HR11 NR1

DONOR HR21 NR2

DONOR HR22 NR2

ACCEPTOR O C

IC -C CB *N HN 1.3476 123.8900 180.0000 114.4700 0.9987

IC -C N CB CA 1.3476 123.8900 180.0000 106.3800 1.5594

IC +N CA *C O 1.3483 117.6500 180.0000 120.4900 1.2287

IC CA C +N +CA 1.5229 117.6500 180.0000 124.1000 1.4523

IC HA C *CA CB 1.1060 109.8600 122.4900 112.4500 1.5594

IC HA C *CA HA2 1.1060 109.8600 -115.6300 107.0500 1.0832

IC CG CA *CB HB 1.5475 115.9500 120.0500 106.4000 1.1163

IC CG CA *CB N 1.5475 115.9500 -125.8100 109.5500 1.481

IC CD CB *CG HG1 1.5384 114.0100 125.2000 108.5500 1.1121

IC CD CB *CG HG2 1.5384 114.0100 -120.3000 108.9600 1.1143

IC CE CG *CD HD1 1.5384 114.0100 125.2000 108.5500 1.1121

IC CE CG *CD HD2 1.5384 114.0100 -120.3000 108.9600 1.1143

IC NZ CD *CE HE1 1.5034 107.0900 120.6900 109.4100 1.1143

IC NZ CD *CE HE2 1.5034 107.0900 -119.0400 111.5200 1.1150

IC CQ CE *NZ HZ 1.3401 123.0500 180.0000 113.1400 1.0065

IC HR11 CQ *NR1 HR12 0.9903 120.6100 171.1900 116.2900 1.0023

IC NR1 NZ *CQ NR2 1.3311 118.0600 178.6400 122.1400 1.3292

IC HR21 CQ *NR2 HR22 0.9899 119.9100 166.1600 116.8800 0.9914

IC CA CB N HN 1.5590 112.2700 73.2100 112.1600 1.0150

IC HB CB N HN 1.1260 106.3500 -169.4700 112.1600 1.0150

IC CG CB N HN 1.5420 109.9800 -52.9500 112.1600 1.0150

IC CD CG CB CA 1.5300 114.7300 -51.9700 112.5700 1.5590

IC HG1 CG CB CA 1.1120 108.2200 -174.7100 112.5700 1.5590

IC HG2 CG CB CA 1.1100 108.8000 71.2800 112.5700 1.5590

IC CD CG CB N 1.5300 114.7300 74.0200 109.9800 1.4810

IC HG1 CG CB N 1.1120 108.2200 -48.7100 109.9800 1.4810

IC HG2 CG CB N 1.1100 108.8000 -162.7200 109.9800 1.4810

IC CD CG CB HB 1.5300 114.7300 -170.4000 107.8500 1.1260

IC HG1 CG CB HB 1.1120 108.2200 66.8600 107.8500 1.1260

IC HG2 CG CB HB 1.1100 108.8000 -47.1400 107.8500 1.1260

IC HA2 CA CB N 1.1130 109.7700 -50.3700 112.2700 1.4810

IC C CA CB N 1.5020 112.0200 -170.7600 112.2700 1.4810

IC HA CA CB N 1.1060 110.2800 64.8000 112.2700 1.4810

IC HA2 CA CB HB 1.1130 109.7700 -166.9900 107.5100 1.1260

IC C CA CB HB 1.5020 112.0200 72.6200 107.5100 1.1260

IC HA CA CB HB 1.1060 110.2800 -51.8300 107.5100 1.1260

IC HA2 CA CB CG 1.1130 109.7700 74.3800 112.5700 1.5420

IC C CA CB CG 1.5020 112.0200 -46.0100 112.5700 1.5420

IC HA CA CB CG 1.1060 110.2800 -170.4600 112.5700 1.5420

IC HD1 CD CG CB 1.1100 110.5200 52.2000 114.7300 1.5420

IC HD2 CD CG CB 1.1050 110.7000 -64.6000 114.7300 1.5420

IC CE CD CG CB 1.5440 110.9100 173.2100 114.7300 1.5420

IC HD1 CD CG HG1 1.1100 110.5200 174.1800 109.6200 1.1120

IC HD2 CD CG HG1 1.1050 110.7000 57.3800 109.6200 1.1120

IC CE CD CG HG1 1.5440 110.9100 -64.8100 109.6200 1.1120

IC HD1 CD CG HG2 1.1100 110.5200 -70.5800 109.6800 1.1100

IC HD2 CD CG HG2 1.1050 110.7000 172.6100 109.6800 1.1100

IC CE CD CG HG2 1.5440 110.9100 50.4200 109.6800 1.1100

IC NZ CE CD HD1 1.4740 110.9300 -41.6000 109.0000 1.1100

IC HE1 CE CD HD1 1.1160 110.6200 80.5100 109.0000 1.1100

IC HE2 CE CD HD1 1.1120 110.2900 -162.2700 109.0000 1.1100

IC NZ CE CD HD2 1.4740 110.9300 73.8100 109.8400 1.1050

IC HE1 CE CD HD2 1.1160 110.6200 -164.0800 109.8400 1.1050

IC HE2 CE CD HD2 1.1120 110.2900 -46.8600 109.8400 1.1050

IC NZ CE CD CG 1.4740 110.9300 -163.5000 110.9100 1.5300

IC HE1 CE CD CG 1.1160 110.6200 -41.3900 110.9100 1.5300

IC HE2 CE CD CG 1.1120 110.2900 75.8200 110.9100 1.5300

IC HZ NZ CE HE1 1.0100 117.1200 147.3900 109.8100 1.1160

IC CQ NZ CE HE1 1.4200 124.2200 -32.8100 109.8100 1.1160

IC HZ NZ CE HE2 1.0100 117.1200 31.5000 108.8500 1.1120

IC CQ NZ CE HE2 1.4200 124.2200 -148.6900 108.8500 1.1120

IC HZ NZ CE CD 1.0100 117.1200 -90.0300 110.9300 1.5440

IC CQ NZ CE CD 1.4200 124.2200 89.7800 110.9300 1.5440

IC O C CA HA2 1.2060 127.0400 -18.0400 108.3300 1.1130

IC O C CA HA 1.2060 127.0400 -132.9000 111.2200 1.1060

IC O C CA CB 1.2060 127.0400 103.1900 112.0200 1.5590

IC NR1 CQ NZ HZ 1.3070 124.7000 -5.3900 118.6600 1.0100

IC NR2 CQ NZ HZ 1.4310 116.0100 177.7300 118.6600 1.0100

IC NR2 CQ NZ CE 1.4310 116.0100 -2.0700 124.2200 1.4740

IC HR11 NR1 CQ NR2 1.0110 120.1600 170.2200 119.2200 1.4310

IC HR12 NR1 CQ NR2 1.0110 120.1600 170.2200 119.2200 1.4310

IC HR11 NR1 CQ NZ 1.0110 120.1600 -6.5600 124.7000 1.4200

IC HR21 NR2 CQ NR1 1.0210 112.5100 3.2800 119.2200 1.3070

IC HR22 NR2 CQ NR1 1.0210 115.1000 130.3300 119.2200 1.3070

IC CE NZ CQ NR1 1.5034 123.0500 180.0000 118.0600 1.3311

IC NZ CQ NR1 HR11 1.3401 118.0600 -178.2800 120.6100 0.9903

IC NZ CQ NR2 HR21 1.3401 122.1400 -174.1400 119.9100 0.9899

PATCHING FIRS LBHN LAST CTER

**RESI DIT +0.000 ! D-3,5 Diodotyrosine**

GROUP

ATOM N NH1 -0.47 ! | HD1 IE1

ATOM HN H 0.31 ! HN-N | |

ATOM CA CT1 0.07 ! | HB1 CD1--CE1

ATOM HA HB 0.09 ! | | // \\

GROUP ! HA-CA--CB--CG CZ--OH

ATOM CB CT2 -0.18 ! | | \ __ / \

ATOM HB1 HA 0.09 ! | HB2 CD2--CE2 HH

ATOM HB2 HA 0.09 ! O=C | |

GROUP ! | HD2 IE2

ATOM CG CA 0.00

GROUP

ATOM CD1 CA -0.115

ATOM HD1 HP 0.115

GROUP

ATOM CE1 CA 0.003

ATOM IE1 IU1 -0.003

GROUP

ATOM CZ CA 0.10

ATOM OH OH1 -0.44

ATOM HH H 0.40

GROUP

ATOM CD2 CA -0.115

ATOM HD2 HP 0.115

GROUP

ATOM CE2 CA 0.003

ATOM IE2 IU1 -0.003

GROUP

ATOM C C 0.51

ATOM O O -0.51

BOND CB CA CG CB CD2 CG CE1 CD1

BOND CZ CE2 OH CZ

BOND N HN N CA C CA C +N

BOND CA HA CB HB1 CB HB2 CD1 HD1 CD2 HD2

BOND CE1 IE1 CE2 IE2 OH HH

DOUBLE O C CD1 CG CE1 CZ CE2 CD2

IMPR N -C CA HN C CA +N O

DONOR HN N

DONOR HH OH

ACCEPTOR OH

ACCEPTOR O C

IC -C CA *N HN 1.3476 123.8100 180.0000 114.5400 0.9986

IC -C N CA C 1.3476 123.8100 180.0000 106.5200 1.5232

IC N CA C +N 1.4501 106.5200 180.0000 117.3300 1.3484

IC +N CA *C O 1.3484 117.3300 180.0000 120.6700 1.2287

IC CA C +N +CA 1.5232 117.3300 180.0000 124.3100 1.4513

IC N C *CA CB 1.4501 106.5200 -122.2700 112.3400 1.5606

IC N C *CA HA 1.4501 106.5200 116.0400 107.1500 1.0833

IC N CA CB CG 1.4501 111.4300 180.0000 112.9400 1.5113

IC CG CA *CB HB1 1.5113 112.9400 118.8900 109.1200 1.1119

IC CG CA *CB HB2 1.5113 112.9400 -123.3600 110.7000 1.1115

IC CA CB CG CD1 1.5606 112.9400 90.0000 120.4900 1.4064

IC CD1 CB *CG CD2 1.4064 120.4900 -176.4600 120.4600 1.4068

IC CB CG CD1 CE1 1.5113 120.4900 -175.4900 120.4000 1.4026

IC CE1 CG *CD1 HD1 1.4026 120.4000 178.9400 119.8000 1.0814

IC CB CG CD2 CE2 1.5113 120.4600 175.3200 120.5600 1.4022

IC CE2 CG *CD2 HD2 1.4022 120.5600 -177.5700 119.9800 1.0813

IC CG CD1 CE1 CZ 1.4064 120.4000 -0.1900 120.0900 1.3978

IC CZ CD1 *CE1 IE1 1.3978 120.0900 179.6400 118.7700 2.1160

IC CZ CD2 *CE2 IE2 1.3979 119.9200 -178.6900 118.7700 2.1160

IC CE1 CE2 *CZ OH 1.3978 120.0500 -178.9800 120.2500 1.4063

IC CE1 CZ OH HH 1.3978 119.6800 175.4500 107.4700 0.9594

**RESI ABC 0.000 ! 1-aminocyclobutanecarboxylic acid**

GROUP

ATOM N NH1 -0.47 ! | HB1

ATOM HN H 0.31 ! HN-N |

ATOM CA CB1 0.16 ! | CB--HB2

GROUP ! | / \

ATOM CB CB2 -0.18 ! CA CG---HG1

ATOM HB1 HA 0.09 ! | \ / \

ATOM HB2 HA 0.09 ! | CD HG2

GROUP ! O=C | \

ATOM CG CB2 -0.18 ! | HD1 HD2

ATOM HG1 HA 0.09 !

ATOM HG2 HA 0.09

GROUP

ATOM CD CB2 -0.18

ATOM HD1 HA 0.09

ATOM HD2 HA 0.09

GROUP

ATOM C C 0.51

ATOM O O -0.51

BOND CA CB CG CB HB1 CB HB2 CB

BOND CD CA C CA N CA CD CG

BOND HG1 CG HG2 CG HD1 CD HD2 CD

BOND HN N C +N

DOUBLE O C

IMPR N -C CA HN C CA +N O

DONOR HN N

ACCEPTOR O C

IC -C CA *N HN 1.3476 123.8100 180.0000 114.5400 0.9986

IC -C N CA C 1.3476 123.8100 180.0000 106.5200 1.5232

IC +N CA *C O 1.3484 117.3300 180.0000 120.6700 1.2287

IC N C *CA CB 1.506 110.41 -123.00 114.69 1.540

IC N C *CA CD 1.506 110.41 120.45 114.69 1.540

IC CA C +N +CA 1.5232 117.3300 180.0000 124.3100 1.4513

IC N CA C +N 1.506 110.41 180.00 115.50 1.3484

IC N CA CB HB1 1.506 117.34 -101.48 116.10 1.081

IC N CA CB HB2 1.506 117.34 25.60 112.20 1.087

IC N CA CB CG 1.506 117.34 138.89 88.14 1.532

IC C CA CB HB1 1.569 114.69 20.83 116.10 1.081

IC C CA CB HB2 1.569 114.69 147.91 112.20 1.087

IC C CA CB CG 1.569 114.69 -99.30 88.14 1.532

IC O C CA N 1.205 112.42 0.00 110.41 1.506

IC O C CA CD 1.205 112.42 129.36 114.69 1.540

IC O C CA CB 1.205 112.42 -129.36 114.69 1.540

IC CD CA CB HB1 1.540 89.25 137.82 116.10 1.081

IC CD CA CB HB2 1.540 89.25 -95.10 112.20 1.087

IC CD CA CB CG 1.540 89.25 17.69 88.14 1.532

IC CD CG CB HB1 1.545 88.94 -136.33 117.70 1.081

IC CD CG CB HB2 1.545 88.94 95.56 111.76 1.087

IC CD CG CB CA 1.545 88.94 -17.64 88.14 1.540

IC CG CD CA N 1.545 88.14 -138.39 117.35 1.506

IC CG CD CA C 1.545 88.14 99.30 114.69 1.569

IC CG CD CA CB 1.545 88.14 -17.69 89.25 1.540

IC HG1 CG CB HB1 1.083 116.75 104.02 114.58 1.081

IC HG1 CG CB HB2 1.083 116.75 -24.09 115.32 1.087

IC HG1 CG CB CA 1.083 116.75 -137.29 89.92 1.540

IC HG2 CG CB HB1 1.078 111.36 -23.75 114.58 1.081

IC HG2 CG CB HB2 1.078 111.36 -151.86 115.32 1.095

IC HG2 CG CB CA 1.078 111.36 94.94 89.92 1.540

IC HD2 CD CA N 1.081 116.10 101.48 118.34 1.506

IC HD2 CD CA C 1.081 116.10 -20.83 113.43 1.569

IC HD2 CD CA CB 1.081 116.10 -137.82 89.55 1.540

IC HD2 CD CG HG1 1.081 117.70 -104.02 114.73 1.083

IC HD2 CD CG HG2 1.081 117.70 23.75 114.33 1.078

IC HD2 CD CG CB 1.081 117.70 136.33 90.52 1.532

IC HD1 CD CA N 1.087 112.20 -25.60 118.34 1.506

IC HD1 CD CA C 1.087 112.20 -147.91 113.43 1.569

IC HD1 CD CA CB 1.087 112.20 95.10 89.55 1.540

IC HD1 CD CG CB 1.087 111.76 -95.56 90.52 1.532

IC HD1 CD CG HG1 1.087 111.76 24.09 114.73 1.083

IC HD1 CD CG HG2 1.087 111.76 151.86 114.33 1.078

IC HN N CA CD 1.018 111.47 70.44 118.34 1.540

IC HN N CA C 1.018 111.47 -159.42 107.57 1.569

IC HN N CA CB 1.018 111.47 -33.53 114.97 1.540

IC CA CD CG HG1 1.540 88.14 137.29 114.73 1.083

IC CA CD CG CB 1.540 88.14 17.64 90.52 1.532

IC CA CD CG HG2 1.540 88.14 -94.94 114.33 1.078

PATCHING FIRS ABCN LAST CTER
